# Tissue- and Time-Specific Expression of Otherwise Identical tRNA Genes

**DOI:** 10.1371/journal.pgen.1006264

**Published:** 2016-08-25

**Authors:** Dror Sagi, Roni Rak, Hila Gingold, Idan Adir, Gadi Maayan, Orna Dahan, Limor Broday, Yitzhak Pilpel, Oded Rechavi

**Affiliations:** 1 Department of Neurobiology, Wise Faculty of Life Sciences and Sagol School of Neuroscience, Tel Aviv University, Tel Aviv, Israel; 2 Department of Molecular Genetics, The Weizmann Institute of Science, Rehovot, Israel; 3 Department of Cell and Developmental Biology, Sackler School of Medicine, Tel Aviv University, Tel-Aviv, Israel; University of California San Diego, UNITED STATES

## Abstract

Codon usage bias affects protein translation because tRNAs that recognize synonymous codons differ in their abundance. Although the current dogma states that tRNA expression is exclusively regulated by intrinsic control elements (A- and B-box sequences), we revealed, using a reporter that monitors the levels of individual tRNA genes in *Caenorhabditis elegans*, that eight tryptophan tRNA genes, 100% identical in sequence, are expressed in different tissues and change their expression dynamically. Furthermore, the expression levels of the *sup-7* tRNA gene at day 6 were found to predict the animal’s lifespan. We discovered that the expression of tRNAs that reside within introns of protein-coding genes is affected by the host gene’s promoter. Pairing between specific Pol II genes and the tRNAs that are contained in their introns is most likely adaptive, since a genome-wide analysis revealed that the presence of specific intronic tRNAs within specific orthologous genes is conserved across *Caenorhabditis* species.

## Author Summary

Regulating the expression levels of each tRNA gene is critical: even “silent” mutations, which do not change protein sequences, but change the identity of the tRNA molecules that deliver the amino acid to the ribosome, can lead to protein misfolding and disease. Dynamic changes in the expression of distinct tRNA gene copies are difficult to measure, because tRNAs are transcribed by Pol III, which cannot transcribe fluorescent reporters such as GFP. We used a reporter system that allows monitoring the activity of individual tRNA gene copies in live *Caenorhabditis elegans* nematodes. Although it is widely believed that tRNA expression is exclusively regulated by intrinsic control elements (A- and B-box sequences), our data revealed that eight tRNA genes, 100% identical in sequence, are distinctively expressed in different tissues, and that they change their expression dynamically. We also found that the expression of tRNAs that reside inside the introns of protein-coding genes is affected by the promoters of the hosting protein-coding genes. In addition, we found a decline in the tRNA expression levels with age, an observation which could be relevant for protein aggregation diseases. Age-related decline in tRNA levels may abrogate co-translational folding, and thus lead to protein misfolding, a hallmark of neurodegeneration.

## Introduction

The availability of mature transfer RNAs (tRNAs) that can deliver amino acids to the ribosome affects protein translation rates [1,2]. Since proteins are co-translationally folded, supplying the right amount of each tRNA is crucial for accurate protein folding [3–8]. Several disorders in which proteins are misfolded arise due to tRNA mutations, alterations in tRNAs levels, and aberrations in tRNA processing or tRNA modifications. For example, abnormal expression of tRNAs was shown to support cancer progression and metastasis [9–11].

Interestingly, the disease phenotypes of many tRNA-based pathologies are tissue specific [12,13]. tRNA-related mutations are of particular clinical interest, in part since such mutations were identified under various neurological conditions (in which protein misfolding is a hallmark of the pathology) [14,15]. For example, brain malformations and microcephaly are associated with tRNA editing [14], Charcot–Marie–Tooth (CMT) syndrome is linked to mutations in several tRNA synthetase genes [16], and the progression of Huntington disease is affected by the depletion of charged tRNA^Gln^ (CUG) [17]. A mutation in a tRNA^Arg^ gene that is expressed specifically in the central nervous system, when combined with the loss of GTPBP2 (a novel binding partner of the ribosome recycling protein), leads to ribosome stalling, and results in neurodegeneration in mice [18]. Although different tissues and cellular processes were shown to require specific tRNA pool compositions [11,19–23], in multicellular organisms precise spatiotemporal regulation of tRNA levels is complex and is currently poorly understood.

Isoacceptors are tRNAs that carry the same amino acid, yet recognize different sets of one or more codons using distinct anti-codon sequences. The presence of multiple codons that code for the same amino acid is often dismissed as redundancy (“the redundancy of the genetic code” [24]). Nevertheless, converging evidence suggests that translation rates, and hence, also co-translational protein folding, are affected by the identity of the specific tRNA isoacceptor that carries the amino acid. These effects arise, at least in part, since isoacceptors differ in their abundance [22,23,25]. Indeed, various so-called “silent” mutations, which do not alter the sequence of the peptide, but change the codon and therefore also the identity of the recruited tRNA isoacceptor, were found to affect protein folding, the deposition of post-translational modifications, and ultimately, they lead to the development of various diseases [7,26–30].

In Eukarya, tRNA isoacceptors can be encoded by multiple nuclear genes, many of which are completely identical in sequence, which are distributed in different genomic locations [31,32]. Is it possible that differential expression of specific tRNA gene copies, which encode for the same tRNA isoacceptor, is consequential? And specifically, could expressing specific tRNA gene copies under specific conditions affect protein translation?

The current dogma holds that the transcription levels of tRNAs that are identical in sequence will be equal, since tRNA expression is controlled by tRNA-intrinsic regulatory elements, known as the A- and B-box, which are contained within the tRNA sequence itself, in the D and TΨP loops [33–36]. In agreement with this assumption, it was previously shown, based on codon usage analysis in several organisms, that the gene copy-number of tRNA isoacceptors correlates with the predicted tRNA levels [22]. In the absence of an alternative explanation, the presence of multiple isoacceptors in the genome was thought to confer robust translation, and to protect the organism from acquiring otherwise detrimental mutations in single-copy tRNA genes. However, examining a tRNA deletion library in yeast revealed that deleting specific copies of a tRNA isoacceptor results in different phenotypes [37]. This work and that of others [38] suggest that multiple isoacceptor genes exist not simply to provide protective redundancy, but rather, that each tRNA gene might be expressed under specific conditions, or at different levels.

tRNA transcription is carried out by RNA polymerase III which binds tRNA intrinsic sites [33,34,36,39]. Although dedicated mechanisms for spatiotemporal regulation of Pol III activity are not well understood, some studies suggest that tRNA genes may be differentially expressed in a time- and tissue-specific manner [13,20,37,40–42]. The gap in knowledge regarding the underlying mechanisms that regulate tRNA transcription stems largely from the requirement for additional methods that enable dynamic visualization of tRNA expression. Since tRNAs are transcribed by Pol III, which can only transcribe short sequences, and since tRNA expression depends on tRNA-intrinsic sequences, the expression of fluorescent reporter proteins (such as GFP) under the control of tRNA promoters is not feasible [34]. The difficulty in measuring tRNA levels is further compounded because tRNA sequences are short, heavily modified, and contain tight secondary structures. Although these difficulties complicate attempts to quantify tRNA levels using probes or sequencing, new tRNA-seq methods are being developed [43–45]. Microarrays have been successfully used to monitor tRNA levels in the past; however, any technique that is based on hybridization or sequence analysis would lack the ability to discriminate between identical or highly similar copies of tRNA genes.

Here we used a new method that allows measuring the levels of mature tRNAs *in vivo*; moreover, it enables one to distinguish between identical tRNA copies. This technique is based on utilizing a nonsense read-through reporter system. Importantly, it permits analyzing the temporal and spatial expression of individual tRNA genes at single-cell resolution in live *Caenorhabditis elegans* nematodes. Using this system, we monitored the expression patterns of eight individual tryptophan tRNA genes that are identical in sequence, and found that each of these genes displays a unique and highly reproducible expression pattern that changes during development and aging. Surprisingly, we discovered that tRNAs that reside within introns of protein-coding genes are regulated by the promoter of the flanking Pol II gene. Genome-wide analysis revealed that intronic and non-intronic tRNAs can be distinguished, based on Pol III occupancy, further suggesting that the unique genomic surrounding affects tRNA expression. In addition, our analysis revealed that specific tRNA genes reside within introns of the same (orthologous) protein-coding genes in different *Caenorhabditis* species, suggesting that pairing between the genomic locations of specific tRNAs and protein-coding genes is adaptive.

## Results

### Measuring tRNA levels in live animals

To study the unique expression patterns of each tRNA gene copy, we focused on tryptophan tRNAs, since all the tryptophan tRNAs in *C*. *elegans* share the CCA anti-codon. Ten of the twelve worms' tryptophan tRNA genes displayed 100% sequence similarity, including their canonical intrinsic Pol III control elements, the A- and B-box sequences. In classical genetic suppressor screens, in which read-through of an amber (UAG) stop codon was assayed, mutations in eight of the ten identical tryptophan tRNA genes were revealed (such mutations are termed “suppressor mutations” or “*sup”*). The found mutations change the CCA tryptophan anti-codon to a CUA amber anti-codon [41,46–48]. The ability of different *sup* mutants to read-through stop codons in specific mutated genes was estimated previously [41,46]. Although such mutants develop properly and are fertile, some sickness does occur since all the amber stop codons in the genome have the potential to be read-through; the sickness manifests itself as slow growth, a smaller brood size, and a restricted range of temperatures in which the mutants can be cultivated (22°C–24°C instead of 15°C –25°C, the *sup* mutants cannot grow in cold temperatures) [49].

**Fig 1:**
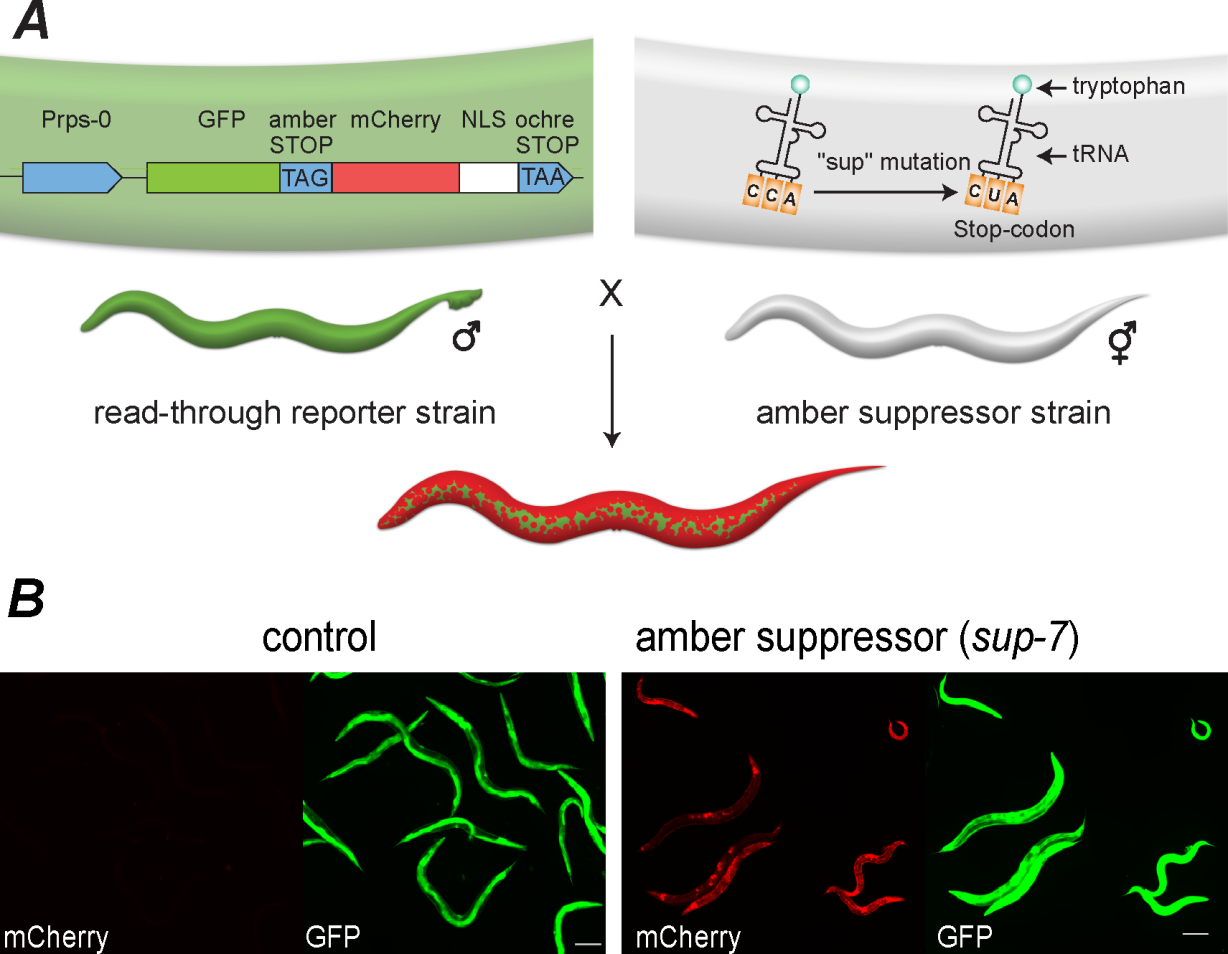
Crossing worms that contain a read-through reporter with tRNA suppressor mutants was used to report tRNA expression. (A) A schematic representation of the tRNA reporter system. To monitor tRNA expression, we created worms that are homozygous for the *sup* mutation, the *smg-2* mutation, and the reporter construct. (B) mCherry expression is a proxy for tRNA levels, and GFP reports the activity of the *rps-0* promoter. Mixed-stage populations of control worms (no read-through activity) and *sup-7* mutants are shown.

To monitor tRNA expression, we crossed the different tryptophan tRNA *sup* mutants with a previously generated worm strain that contains a fluorescent reporter (this strain has been used as a “synthetic biology” tool that allows incorporating non-typical amino acids into proteins [50]); henceforth, it will be referred to as the “Read-through Reporter Strain” (abbreviated as RRS). The RRS worms contain an integrated, low-copy transgene that expresses a fused product of the GFP protein and a nuclear localization signal (NLS)-tagged mCherry protein, which are separated by an amber stop-codon (Fig 1A). Expression of these proteins is controlled by the *rps-0* promoter, which is ubiquitously expressed in the soma [50]. These transgenic worms display bright green fluorescence in all somatic tissues (no detectable expression is seen in the germline); however, red fluorescence cannot be detected at all, since translation of the mCherry protein is blocked due to the presence of the early amber stop codon. Thus, owing to the transgene’s design, the intensity of red fluorescence serves as a proxy for the prevalence of read-through events. In this way, it effectively reports the expression levels of the suppressor tRNA (Fig 1) (in a related study, read-through of a stop codon was used to report the suppression activity that an *in-vitro*-transcribed synthetic tRNA can induce in cell culture [38]). To allow proper translation of the reporter gene, these worms were engineered to contain a mutation in the *smg-2* gene, which disables the nonsense-mediated decay (NMD) pathway, which would otherwise lead to mRNA degradation upon premature stop codon recognition [51]. Each of the suppressor tRNA strains was backcrossed multiple times to the RRS worms to eliminate artifacts that could stem from background mutations. In summary, we established a reporter system in which *mCherry* expression is a proxy for the activity of specific tRNA genes.

### tRNA genes that are identical in sequence exhibit unique expression patterns

**Fig 2:**
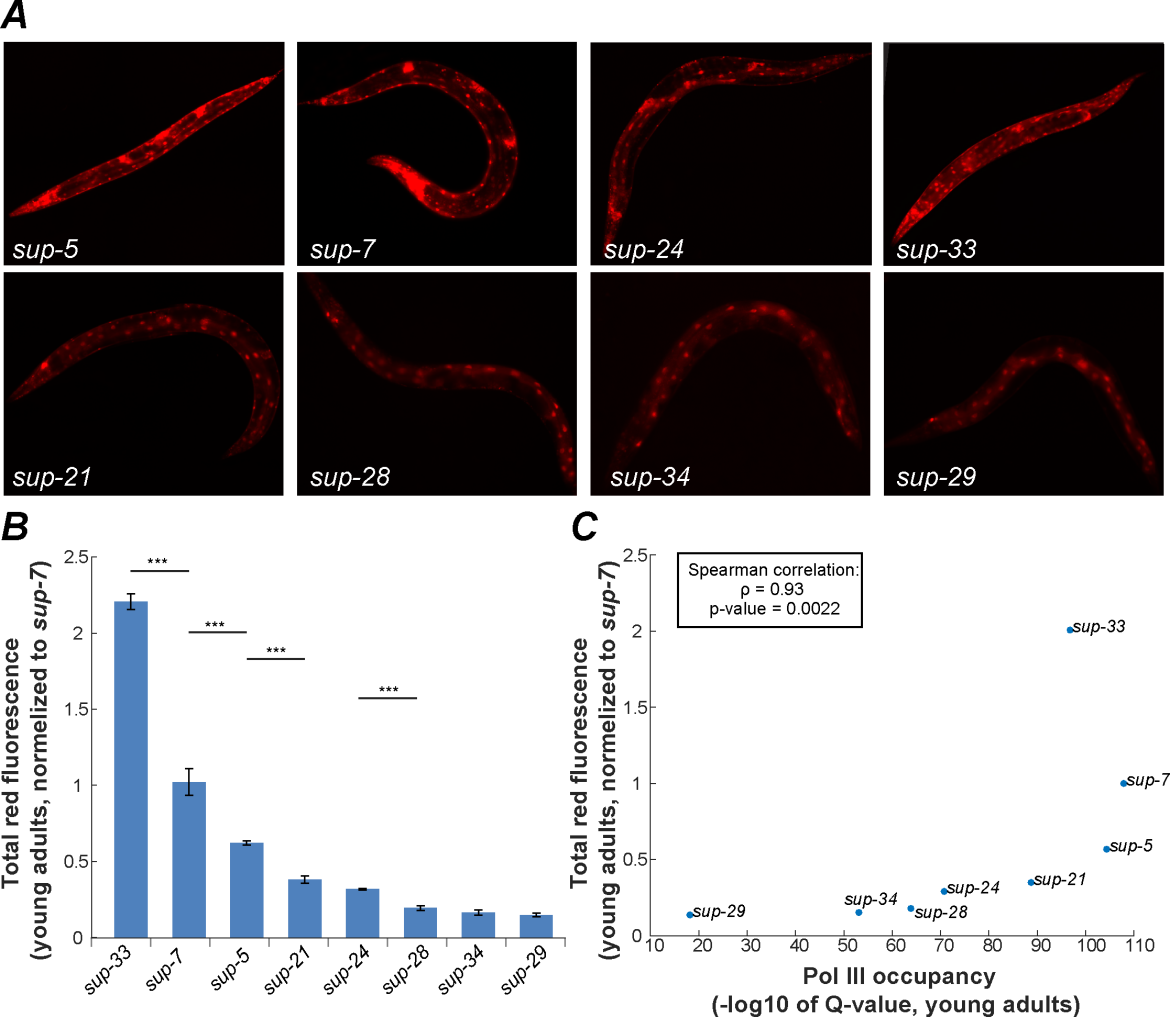
Copies of tryptophan tRNA genes display different expression patterns. (A) Representative images of young adult worms; all eight tryptophan tRNA reporter strains are displayed (90/90 worms exhibited the same expression patterns). mCherry expression is a proxy for the expression of specific tryptophan tRNA genes. Images were taken under exposure conditions that also allow detecting expression in tissues where the tRNA levels are relatively low. (B) Quantification of total worm mCherry fluorescence. All strains were analyzed when the worms were the same age (young adults), using the same exposure parameters. Shown are averages of means, ± SEM, normalized to the expression levels detected in the *sup-7* strain. Three experiments were conducted, and 30 worms of each strain were scored in each experiment. *** p-value<0.001 (C) The results that were obtained using the fluorescent reporter were plotted against the Chip-seq measurements of pol III occupancy [52].

**Fig 3:**
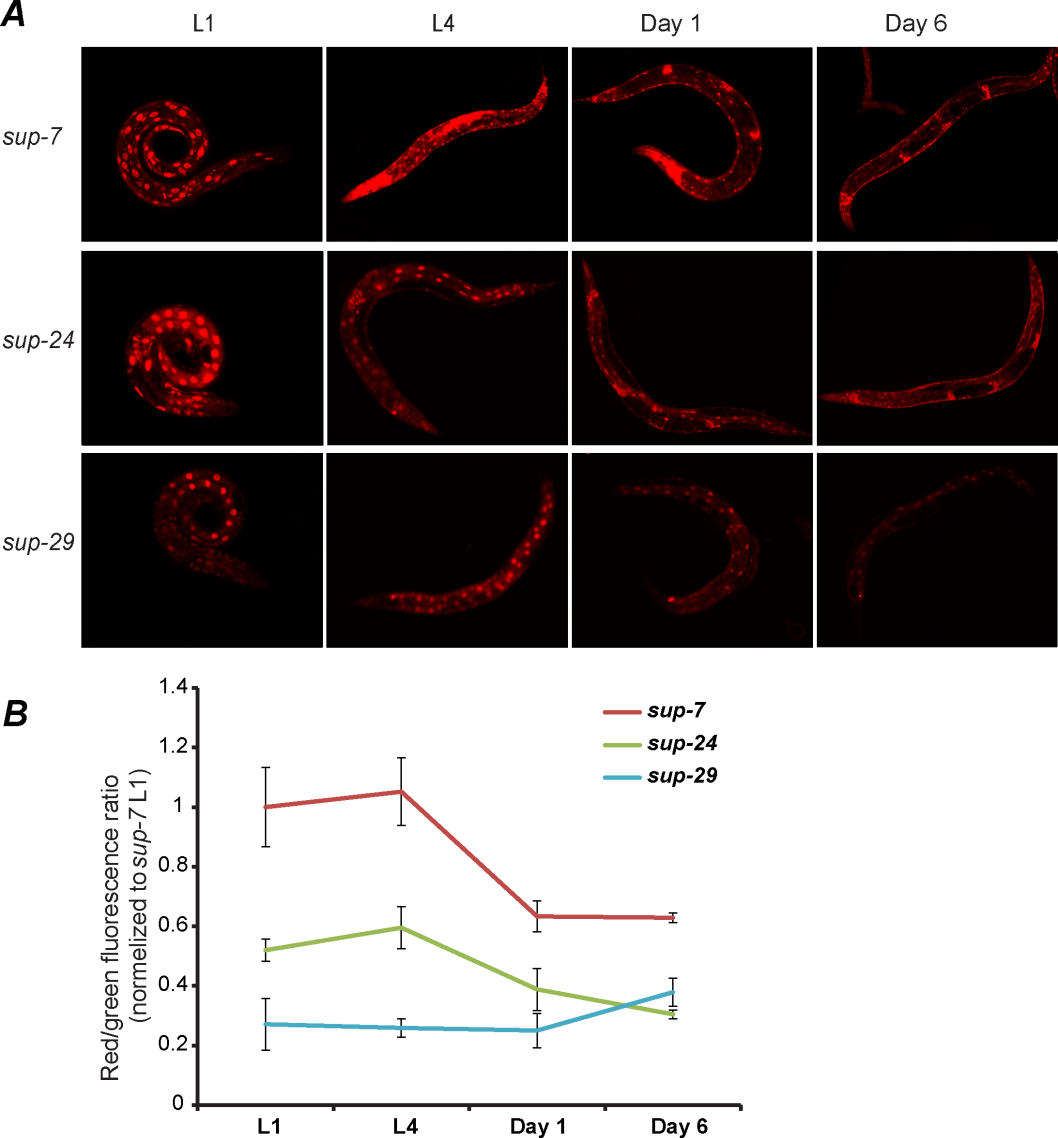
Differential expression of tryptophan tRNAs in *C*. *elegans* throughout time. (A) Representative images of three tryptophan tRNA reporter strains at various stages and ages. Images were taken under exposure conditions allowing optimal visualization of the expression patterns. (B) Quantification of total worm mCherry fluorescence was normalized to total worm GFP fluorescence, in the tRNA reporter strains, at different stages and ages. Measurements of the fluorescence in the different strains were taken at the same time using the same exposure parameters. Shown are averages of means, ± SEM; three experiments were conducted, N = 30 worms from each strain in each stage per experiment.

Our reporter allows dynamic detection of tRNA expression, and since the mCherry reporter protein is tagged with an NLS that restricts its expression in space (to the nucleus), individual cells in which the tRNAs are expressed can be identified. The spatial expression patterns that are reported for specific tRNAs using this transgenic system are highly replicable. For example, whereas the *sup-7* tRNA gene was found to be expressed in all somatic tissues, nuclear mCherry expression was much stronger in neurons and in the vulva in every measured young adult (90/90 worms, Fig 2A). The upstream GFP protein, which simply reports the activity of the *rps-0* promoter, is expressed in the cytoplasm of all somatic cells, as expected. We used this system to compare the spatial and temporal expression patterns of the eight previously identified tryptophan tRNA suppressors [41], which have identical sequences. Importantly, we detected dramatic differences in the genes’ expression profiles (Fig 2A and 2B, Table 1). We compared, as a negative control, the expression patterns of different alleles of *sup-7* and *sup-*5 (termed *sup-7*, *sup-7B*, *sup-5*, and *sup-5B*), which were isolated independently in different labs [41,49]. Although different *sup* mutants exhibited distinct spatial and temporal expression patterns (Table 1 and Figs 2 and 3), the two *sup-7* alleles and two *sup-*5 alleles were found to be expressed similarly, thus supporting the significance of the differences that were found between the expression patterns of different tRNA genes (S1 Fig).

**Table 1:** Tissue-specific expression of tryptophan tRNAs in *C*. *elegans*. Summary of tissue-specific expression of the eight identical tryptophan tRNA genes. The number of Vs indicates the relative fluorescence intensity. Blind scoring of the expression patterns (by 3 testers) was determined using a confocal microscope.

Strain	Stage	Neurons	Muscles	Hypodermis	Intestine	Vulva and Uterus	Spermatheca	Main visible expression
***sup-7***	L4	VVVV (pan neuronal)	VVV	VVV	VV	VVV	VVV	Head and vulva
** **	Young Adult	VVV	VVV	VV	-	VVV	VVV	Loss of intestinal expression and body neurons in adults
***sup-5***	L4	VVVV (pan neuronal)	VVV	VVV	VV	VVV	VVV	Head and vulva
** **	Young Adult	VVV	VVV	VV	-	VVV	VVV	Loss of intestinal expression and body neurons in adults
***sup-33***	L4	V	VVV	VVVV	VV	VVV	VV	Very weak neuronal expression; strong expression in the hypodermis, muscles and vulva
	Young Adult	-	VVV	VVVV	VV	VVV	VV	Stable expression throughout the transition to adulthood
***sup-24***	L4	VV (pan neuronal)	VVV	VVV	VVV	VV	VVVV	Ubiquitous expression in all tissues
** **	Young Adult	V (pan neuronal)	V	VV	V	VV	VVVV	Loss of intestinal expression and weak muscle expression in adults
***sup-21***	L4	-	VV	VV	VVV	V	VV	Intestine
** **	Young Adult	-	V	V	VV	V	VV	intestine
***sup-28***	L4	-	VV	VVV	VVV	VV	VV	Intestine
*** ***	Young Adult	-	-	VV	V	V	V	The adult displays very weak expression in all tissues
***sup-34***	L4	V	V	VV	VVV	V	-	Intestine
** **	Young Adult	-	-	V	VV	V	-	Barely visible expression in adults
***sup-29***	L4	-	V	-	VVV	V	-	Intestine
** **	Young Adult	-	-	-	VV	-	-	Intestine

Each tRNA gene displayed stereotypic and highly replicable expression patterns. For example, in young adults, *sup-29* is expressed predominantly in the intestine, without any visible expression in neurons. At the same stage, *sup-7* and *sup-5* are predominantly expressed in neurons and in the vulva (Table 1 and Fig 2). However, in L4, *sup-7* is ubiquitously expressed in all somatic tissues. Thus, during the transition to adulthood, *sup-7* exhibits an overall decline in somatic expression and in particular, a loss of intestinal expression (Fig 3A and 3B, L1 or L4 vs. day 1 or day 6, p-value < 0.01, Table 1). On the other hand, *sup-29* remains predominantly intestinal during development and adulthood and is lowly expressed in all stages. *sup-24* is ubiquitously expressed in all larvae stages, but loses its expression in the intestine and in most muscles during the transition to adulthood (Fig 3A and 3B, L4 vs. day 6, p-value < 0.01, Table 1).

The read-through system is currently the only method that enables measuring tissue-specific expression of identical tRNA genes. Although any sequence-based analysis (e.g. sequencing, microarrays) cannot distinguish between products of similar tRNA genes, when ignoring tissue-specificity, approximation of the total expression levels of identical tRNAs can be achieved by analyzing the chromatin composition in the vicinity of the tRNA genes, or specifically by measuring pol III occupancy (see next). To compare our method to alternative whole-worm tRNA-measurement techniques, we first ranked the overall (total worm) suppressor activity of the different tryptophan tRNAs using our reporter (Fig 2B). In young adults, *sup-33* was found to be the strongest suppressor, based on whole-worm red fluorescence intensity (Fig 2B), and the intensity of the other suppressor tRNA genes was ranked such that *sup-7*>sup-5>sup-21>*sup-24*>sup-28>*sup-34*>*sup-29* (*sup-29* is the weakest suppressor). As an alternative method for assessing the whole-worm tRNA expression levels, we analyzed the Pol III occupancy of the different *C*. *elegans* tRNA genes [53]. Importantly, we found a very strong rank correlation (r = 0.93, p-value = 0.0022) between the tRNA expression levels, as predicted by Pol III binding, and the levels that were detected by our fluorescence reporter system (both measurements were conducted using young adult worms, Fig 2C). This consistency strengthens the validity of our fluorescence-based method.

We also examined whether the presence of certain histone modifications, that are generally associated with transcription or repression of tRNA genes [19,54], correlates with the expression patterns that our read-through assay reported (S2 Fig). When averaging the signal of the H3K4me3 modification (associated with transcription) in the vicinity of 609 tRNA genes, we detected a striking enrichment in positions -500 to -100 compared to the first mature nucleotide of the tRNAs. In contrast, when we examined the H3K4me3 signatures in the vicinity of individual tRNA genes, the signal was noisy and incoherent (S2 Fig). We detected a moderate rank correlation between levels of H3K4me3 (r = 0.6, p-value = 0.13) and the levels of tRNAs expression as measured by our reporter, yet the correlation was not statistically significant, potentially due to the small number of reporters examined (S2A Fig). When examined across all 576 tRNA genes (for which both measurements exist), we found significant, but only moderate linear correlation between pol III occupancy and H3K4me3 levels (Spearman correlation = 0.2883, S2B Fig). Similar qualitative results were achieved for the H3K27ac modification (Spearman correlation = 0.0978, S2B Fig). Thus, in summary, based on the comparisons to our reporter assay, and the weak correlation between Pol III binding levels and the presence of these modifications, Pol III binding is a good proxy for estimating the expression levels of individual tRNA genes, while H3K4me and H3K27ac levels are not predictive when estimating the expression levels of the measured tryptophan tRNA genes (S2 Fig).

Our data are also in agreement with classical reports obtained before the development of fluorescent reporters, which were based on the suppression intensities of different amber mutations [47,55]. However, certain expression patterns (for example, the intestinal expression shown in Fig 2 and Table 1) have not been noted in the past, most likely due to the insufficient resolution of the mutant suppression systems that were used [41]. Moreover, for similar technical reasons, temporal differences in the tRNA expression patterns were not detected at all in suppression screens. Such changes over time, and in particular the decline in expression which is seen in older worms, are important, since they could be related to known pathology-associated protein misfolding and aggregation defects, and in particular, to neurodegeneration [8,14,17,18].

**Fig 4:**
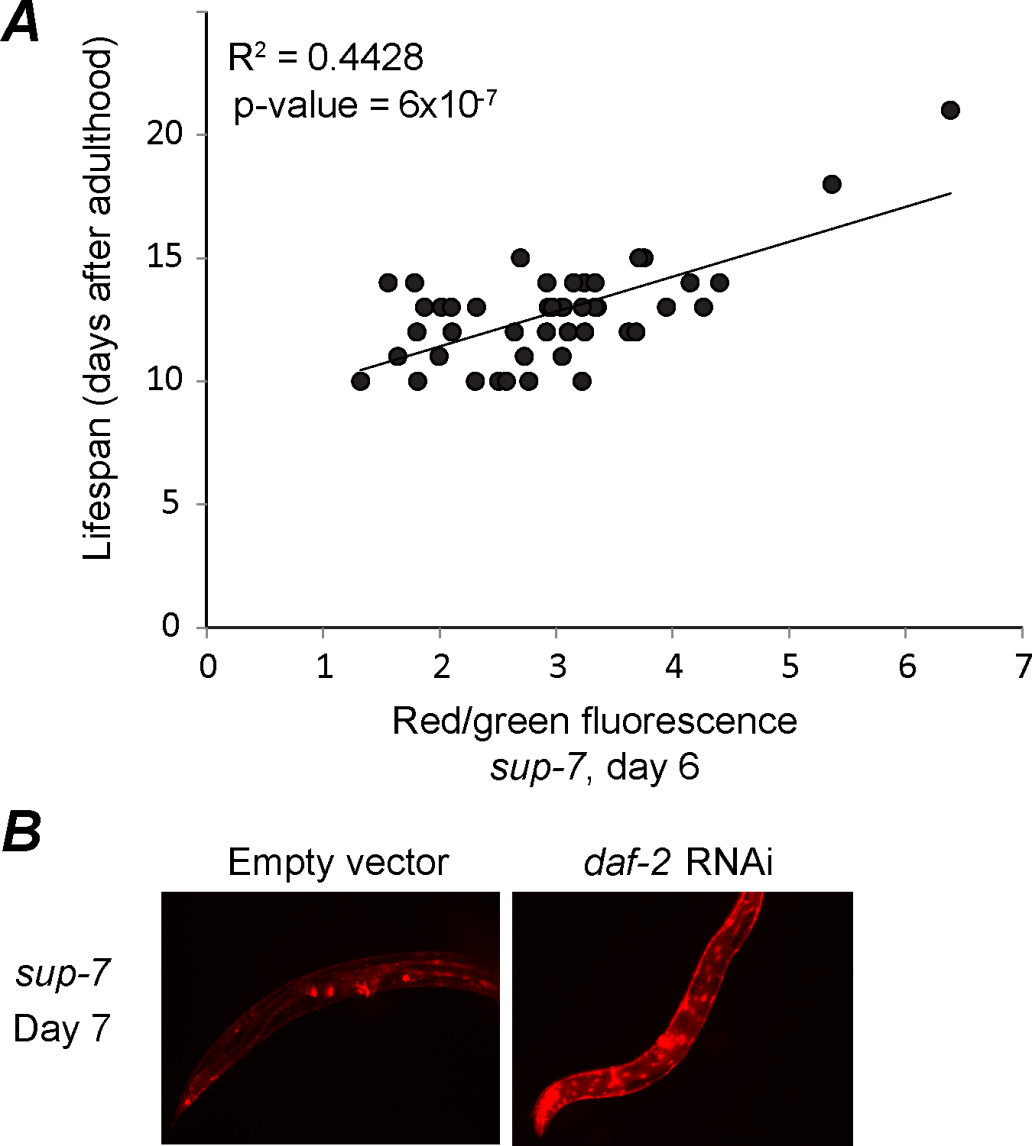
*Sup-7* tRNA expression predicts the worms' lifespan. (A) The intensity of mCherry fluorescence (normalized to GFP) in *sup-7* worms at day 6 (X-axis) correlates with the remaining lifespan of the same worm (Y-axis, two tailed p-value for correlation significance). (B) Shown are representative images of day 7 worms that were fed with bacteria that induce *daf-2* RNAi (right panel) or with bacteria that express an empty RNAi vector (control, left panel).

The expression levels of a few genes such as *sod-3 or ugt-9*, which were found to change monotonically with aging, served in the past as proxies to assess the worms’ biological age [56]. Since the expression of *sup-7* declines with adulthood, we hypothesized that its expression levels in a chronologically synchronized population could reflect the true physiological age and consequently the remaining lifespan of individuals. To test this hypothesis, we tracked individual worms and measured the correlation between *sup-7* expression levels at day 6 of adulthood (about 50% of these worms’ lifespan) and their remaining lifespan. Interestingly, we found that higher levels of *sup-7* at day 6 were correlated with a longer lifespan (Fig 4A). We also found that worms that were treated with *daf-2* RNAi, a treatment that is known to increase lifespan [57], displayed a higher expression of *sup-7* in adulthood in comparison with worms treated with an empty RNAi vector (Fig 4B). While these correlations that we detected do not imply causality, it would be intriguing in future studies to test whether the increased longevity of worms that express high levels of tRNAs stems from their ability to properly fold proteins at an old age.

In summary, we found striking variations in the expression patterns of tryptophan tRNA genes that are identical in sequence. The different *sup* genes are expressed in different tissues, times, and strengths, and aging worms show decreased tRNA expression.

### Intronic tRNAs are regulated by the promoter of their host gene

Despite their sequence identity, the different tryptophan tRNA gene copies are differentially expressed, and therefore, additional control elements (apart from the known regulatory A- and B-box sequences [34]), which reside outside of the tRNA sequence, must affect tRNA expression. Since the tryptophan tRNA genes were found to be expressed in specific tissues, we searched for Cis-control elements that can confer tissue specificity, similarly to Pol II promoters. Seven out of twelve tryptophan tRNAs, and 47% of all the tRNAs in *C*. *elegans* reside in a well-defined genomic environment–within an intron of a protein-coding gene (hereafter referred to as “intronic tRNAs”, see S3 Fig and S1 Table). We reasoned that a systematic dissection of the genomic context of an intronic tRNA could be achieved, since the flanking (hereafter referred to as “host”) gene is a discrete genomic entity with annotated functional regulatory elements. Therefore, although in theory, multiple different mechanisms and control elements could affect tRNA expression (both proximal and distant elements), we based our examination on the hypothesis that a promoter of a host protein-coding gene could influence the transcription of an embedded intronic tRNA, and consequently lead to tissue-specific tRNA expression.

**Fig 5:**
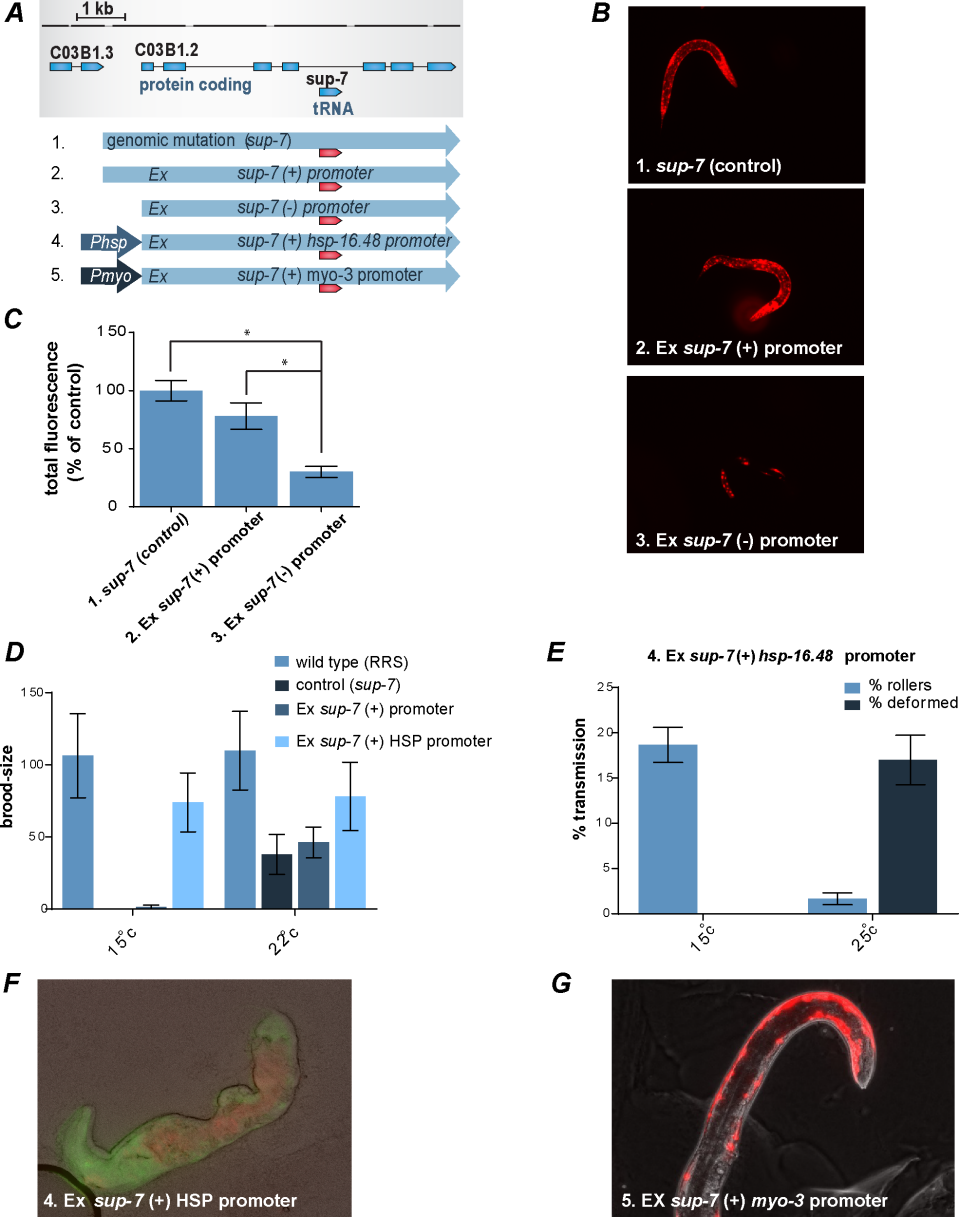
The promoter of the host gene is required for proper tRNA expression. (A) Schematic representation of the *sup-7* constructs. All the constructs that are shown here include the *sup-7* suppressor tRNA. (1) A *sup-7* mutation in the genome (*sup-7*). (2) A construct that includes the *C03B1.2* gene, including its native promoter (“*sup-7* (+) promoter”). (3) A construct that includes only the *C03B1.2* gene, without a promoter (“*sup-7* (-) promoter”). (4) A construct in which the endogenous promoter of the *C03B1.2* gene was swapped with the *hsp-16.48* promoter (“*sup-7* (+) HSP promoter”). (5) A construct in which the endogenous promoter of the *C03B1.2* gene was swapped with a *myo-3* promoter (“*sup-7* (+) *myo-3* promoter”). (B) The expression pattern of a tRNA in L1 transgenic worms that express an extrachromosomal "*sup-7* (+) promoter" construct (middle panel) is highly similar to the expression pattern displayed by mutants that harbor the genomic suppression mutation (upper panel). On the other hand, L1 transgenic worms expressing extrachromosomally the "*sup-7* (-) promoter" construct exhibit aberrant expression (lower panel) and sterility (Table 2), and therefore, are significantly different from the *sup-7* (control) strain. No expression was found when construct 6 was injected (Table 2). (C) Quantification of the total mCherry fluorescence levels normalized to *sup-7* control worms (n = 15; error bars are s.d., * p < 0.01). (D) At 15°C only worms that do not have a *sup-7* mutation (the RRS strain) and the worms that express the “*sup-7* (+) HSP promoter” transgene are fertile. Worms harboring a genomic mutation in *sup-7* (control, *sup-7*) as well as transgenic worms that express the “*sup-7* (+) promoter” transgene are sterile. At 22°C all strains are fertile (an average of 3 experiments ± SEM, the brood size of 5 worms was monitored in each experiment). (E) Transgenic worms expressing the “*sup-7* (+) HSP promoter” transgene are healthy at 15°C and sick at 25°C. Sickness is manifested as a 10-fold reduction in the number of transgenic progeny and the appearance of severe deformations (an average of 3 experiments ±SEM, N = 100 per experiment). *rol-6* was used as a co-injection marker. (F) A deformed worm expressing the “*sup-7* (+) HSP promoter” transgene at 25°C. Shown are mCherry (red), GFP (green), and phase (gray) images. (G) The few transgenic worms that survives and expressed the “*sup-7* (+) *myo-3* promoter” transgene displayed expression of *sup-7* in muscles and were sterile (Table 2).

As a proof-of-principle tool for studying the possible influence of the host gene’s promoter on tRNA expression, we examined the intronic tRNA *sup-7*. *sup-*7 resides in the 3^rd^ intron of the protein-coding gene, *C03B1*.*2*, and based on our reporter assay, it possesses strong read-through activity. We injected the *C03B1*.*2* gene into the reporter strain worms (RRS). The injected extrachromosomal array contained the full sequence of the *C03B1*.*2* protein-coding gene, including the intron in which the mutated, read-through-inducing *sup-7* tRNA gene resides. We cloned various promoters upstream of the injected *C03B1*.*2* gene (Fig 5A and Table 2). Interestingly, we found that an injected construct that contains the *C03B1*.*2* gene, but does not contain *C03B1*.*2*’s endogenous promoter, does not enable the recovery of viable lines (in RRS and N2 worms, Table 2). We also found that the injected animals produced F1s that were extremely sick and exhibited aberrant expression that was very different from the expression of the control *sup-7* worms with the genomic mutation (as reported by a read-through of mCherry, Fig 5B and 5C, Table 2). In contrast, injecting a construct in which *C03B1*.*2*’s expression was controlled by the gene’s native promoter enabled the recovery of multiple viable lines, and the transgene-derived tRNA displayed expression patterns that were very similar to the expression patterns that were displayed by the genomic *sup-7* tRNA *(*Fig 5B and 5C, Table 2 and S4 Fig). These results suggest that the host gene’s promoter affects the expression of the intronic tRNA, and that in the absence of this regulation the tRNA becomes lethal, perhaps due to ectopic expression. In the past, a similar vector that expresses a promoter-less *sup-7* tRNA was used as a lethal co-injection marker [58].

**Table 2:** The expression of *sup-7* tRNA is influenced by the promoter of the host gene, *C03B1*.*2*. The read-through reporter strain (RRS) or N2 worms were injected with the indicated constructs (the construct number corresponds to its number in Fig 5). We screened all the progeny for either red fluorescence (indicating *sup-7* expression) or for the presence of the corresponding co-injection marker. Viable lines that express the *sup-7* tryptophan tRNAs ("*sup-7* (+) promoter") were obtained by cultivating the injected worms at 22°C. Worms that were injected with the "*sup-7* (+) HSP" promoter were cultivated at 15°C.

Injection into RRS^a^or N2^b^	N^d^	No. of F1	F1s’ sterility	Number of viable lines
2. *C03B1*.*2* gene including its promoter: "*sup-7* (+) promoter"^a^	65	37	-	5
3. *C03B1*.*2* gene without its promoter: *"sup-7* (-) promoter" ^a^	83	19	+	
4A. *Phsp-16*.*48*::*C03B1*.*2*: "*sup-7* (+) HSP promoter" ^a^^,^^c^	15	20	-	3
4B. *Phsp-16*.*1*:*C03B1*.*2* (wt-*sup-7*): "*wt-sup-7*(+) HSP promoter" ^a^^,^^c^	22	30	-	4
5. *Pmyo-3*::*C03B1*.*2*: "*sup-7* (+) *myo-3* promoter" ^a^	79	7	+	
6. *Punc-122*::*C03B1*.*2*: "*sup-7* (+) *unc-122* promoter" ^a^	41	0		
7. *sup-7* including only *C03B1*.*2’s* intronic region: “intron only” ^a^	60	3	+	
2. *sup-7* (+) promoter ^b^^,^^c^	40	25	-	5
3A. *sup-7* (–) promoter ^b^^,^^c^	35	0		
3B. *sup-7* (–) promoter ^b^^,^^c^(2.5ug)	25	0		
3C. *sup-7* (–) promoter ^b^^,^^c^ (0.5ug)	25	3^e^	-	

^a^injected into RRS worms (5ng/μl construct + 95ng/μl ladder)

^b^injected into N2 worms (5 ng/μl construct + 10 ng/μl rol-6 + 85ng/μl ladder)

^c^ *rol-6(su1006)* was used as a co-injection marker

^d^ The number of injected animals.

^e^ The mutated *sup-7* did not form part of the extrachromosomal array (EX), as validated by PCR.

To further study the effect of the host gene’s promoter on the expression of the intronic tRNA, we took advantage of the fact that worms that express a read-through-inducing mutated *sup-7* are sterile at 15°C [49]. We generated transgenic worms in which the expression of the *C03B1*.*2* gene (that contained a mutated *sup-7* copy) is controlled by the heat shock promoter, *hsp-16*.*48*, which suppresses transcription at temperatures that are lower than 20°C [59]. We found that at 15°C *sup-7*’s sterility was suppressed, and that the recovery of multiple viable lines from the injected animals was enabled. Thus, the *hsp-16*.*48* promoter inhibits the expression of the intron-residing, Pol III-controlled tRNA *sup-*7 (Table 2). We quantified the transgenic worms’ brood size and compared it to the brood size of RRS worms, in which *sup-7* was not mutated (and therefore, no read-through takes place). At 15°C, RRS worms and the worms in which *sup-7* expression was controlled by the heat shock promoter (*sup-7* (+) HSP promoter) displayed similar brood sizes (Fig 5D). In contrast, RRS worms that contained a mutation in the genomic *sup-7* gene, and RRS worms that were injected with a construct in which the expression of the *C03B1*.*2* gene was controlled by its native promoter (*sup-7* (+) native promoter) did not survive at 15°C or became completely sterile. Upon changing the temperature to 25°C, conditions under which the *hsp-16*.*48* promoter produces “leaky” transcription, “*sup-7* (+) HSP promoter” worms exhibited strong deformation and sterility. As an additional control, we also generated transgenic RRS worms that express an extrachromosomal *hsp-16*.*48*-controlled *C03B1*.*2* gene (termed "wt-*sup-7*(+) HSP promoter") containing an intronic wild-type *sup-7* tRNA gene that does not lead to read-through. We found that transferring these worms from 15°C to 25°C was not at all toxic (and no deformations were observed). These results strengthen the hypothesis that ectopic expression of *sup-7* is toxic (Fig 5E and 5F), and that the host gene’s promoter enables the expression of intronic tRNAs to be restricted.

Similarly to the results that were obtained with the promoter-less construct, and the construct that contained the *hsp-16*.*48* promoter at 25°C, we found that expressing *C03B1*.*2* under two different endogenous promoters, *Pmyo-3* (drives expression in muscles) and *Punc-122* (drives expression in coelomocyte), was highly toxic and no viable line could be obtained, despite multiple injection efforts (79 worms were injected with the *Pmyo-3*::*C03B1*.*2* construct and 41 worms were injected with the *Punc-122*::*C03B1*.*2* construct, Table 2). Finally, among all the worms that were injected with the *Pmyo-3*::*C03B1*.*2* construct, we found only 2 worms (out of a total of 7 F1s that survived) that expressed *sup-7* in muscles (Fig 5G, Table 2).

In summary, our promoter analysis supports a model whereby the expression of an intronic tRNA is affected by the expression of the host gene, and specifically, whereby the Pol II promoter can restrict intronic tRNA expression. This conclusion is based on three lines of evidence (Fig 5, Table 2 and S4 Fig): 1) Proper expression of the intronic *sup-*7 tRNA depends on the presence of the promoter of the *C03B1*.*2* “hosting” gene in the expression vector. In the absence of the gene’s promoter, the tRNA is ectopically expressed, and the enhanced read-through activity leads to lethality and sterility (a promoter-less *sup-7* tRNA served as a lethal co-injection marker in the past [58]). 2) Swapping the endogenous promoter of *C03B1*.*2* with a heat shock promoter suppresses *sup-*7’s sterility at cold temperatures. 3) Swapping the endogenous promoter of the *C03B1*.*2* flanking gene with *myo-3* or *unc-122* promoters is similarly lethal (probably as a result of ectopic expression during development, although this cannot be tested, since the animals die). In the 2 animals that survived and displayed tRNA expression in adulthood, among the animals that were injected with *Pmyo-3::C03B1.2*, the tRNA expression was most notable in muscles, where the *myo-3* promoter is known to promote expression.

### A global analysis of the expression and conservation of intronic tRNAs across nematode species

**Fig 6:**
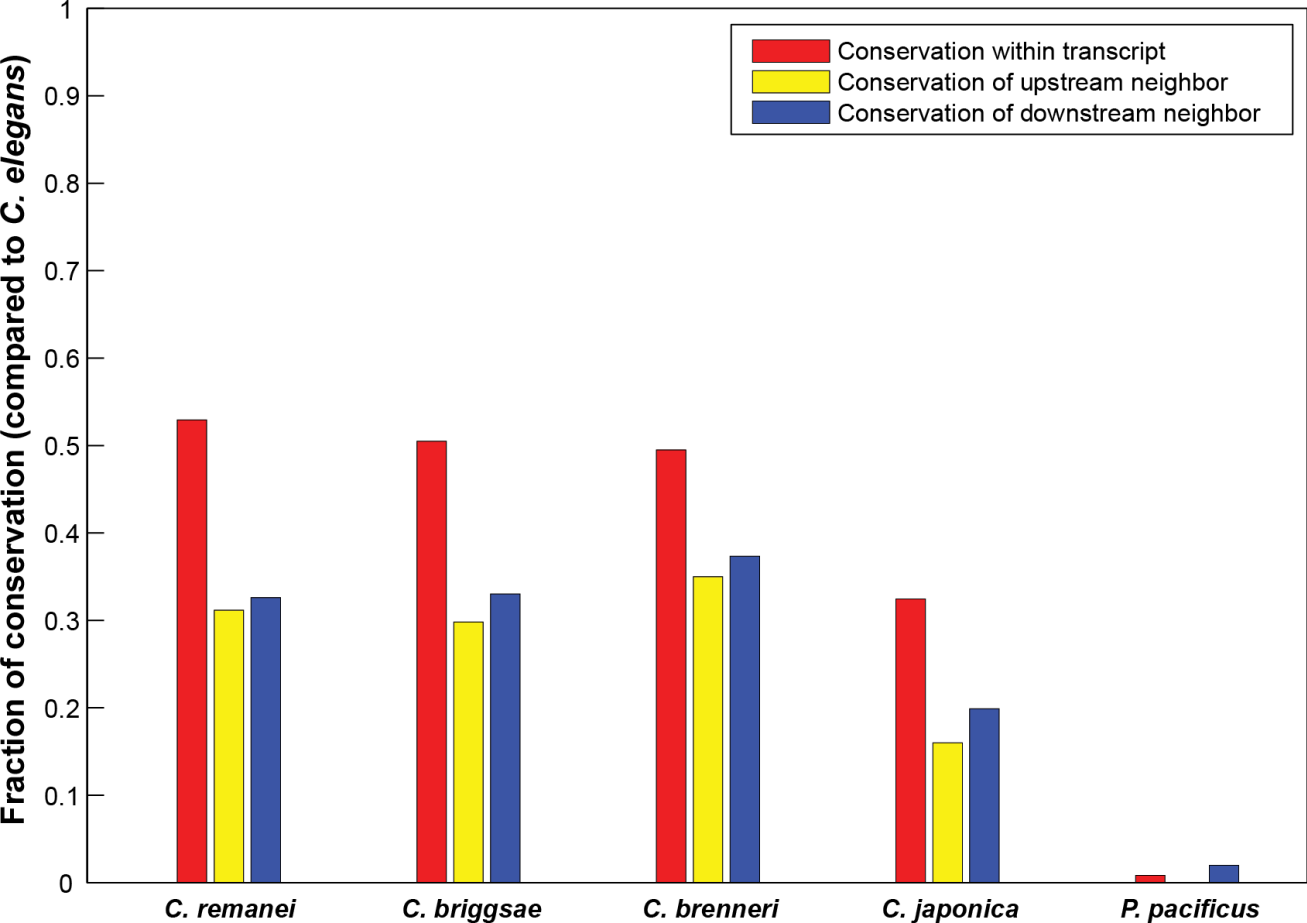
Conservation of the pairing between tRNAs and hosting or neighboring protein-coding genes among nematodes. Shown are the fractions of conserved genomic architectures in different nematodes in comparison to *C*. *elegans*. Each bar denotes the conservation of localization of a given tRNA type (anticodon) with respect to either the tRNA-hosting gene or the adjacent protein-coding genes. Shown are the fractions of conservation of a given anticodon within the transcripts of a specific hosting protein-coding gene (red bars), and the conservation of the adjacent upstream (yellow bars) or downstream (blue bars) protein-coding genes in the vicinity of a given tRNA type (anticodon). The yellow and blue bars were generated based on all the individual tRNA genes that were not localized within the transcripts of protein-coding genes.

Based on the observation that the promoter of the host gene of *sup-7*, *C03B1*.*2* is required for proper regulation of the *sup-*7 tRNA, we looked for a global genomic signature that distinguishes between intronic and non-intronic tRNAs. In total, 44% of the 609 tRNA coding genes in the genome are present within the introns of protein-coding genes (S3 Fig). This percentage of intron residing genes is not significantly different than the fraction expected by chance given the total proportion of introns in the entire genome (p-value = 0.566, chi-square). We then tested whether the presence of specific tRNA genes within introns of specific protein-coding genes is conserved among *Caenorhabditis* species. For this purpose, we analyzed conservation among nematode species of co-occurrences (pairing) of specific intronic tRNA types and specific hosting protein-coding genes. We repeated the classification of tRNAs into intronic and non-intronic ones in four additional *Caenorhabditis* species (*C*. *brenneri*, *C*. *briggsae*, *C*. *japonica*, and *C*. *remanei*), and in the more distant *Pristionchus pacificus* species. Our analysis revealed that 32%-52% of the specific tRNA-host gene pairs that exist in *C*. *elegans* are conserved as pairs among the other *Caenorhabditis* species (red bars in Fig 6), whereas the distant *Pristionchus pacificus* exhibited only a negligible degree of conservation (Fig 6). As a control, we repeated the same analysis for the 211 pseudo tRNA genes that exist in the *C*. *elegans* genome, of which 95 genes reside in introns. We then examined whether these pairs are conserved in *C*. *briggsae* and *C*. *remanei* (there are no predicted pseudo tRNA genes for *C*. *brenneri* and *C*. *japonica*). Interestingly, we found no conservation at all when comparing pseudo tRNAs and protein-coding gene pairs. This complete lack of conservation cannot be trivially attributed to the lower degree of similarity in the composition of nematodes’ pseudo tRNA pools (in terms of gene copy numbers), since the pseudo tRNA pool of *C*. *elegans* is highly similar to that of both *C*. *remanei* (Spearman *ρ* = 0.44; P-value = 0.016) and *C*. *briggsae* (Spearman *ρ* = 0.37; P-value = 0.042). As an alternative null model to check the significance of the conservation of tRNAs within introns we also compared pairing between specific intronic tRNAs and hosting protein-coding genes to the pairing between non-intronic tRNAs and adjacent protein-coding genes, while taking into account the distance between the tRNAs and the protein-coding gene (Fig 6 and S5 Fig). We found that intronic-tRNAs and their specific hosting protein-coding genes are more conserved as pairs than pairs of non-intronic tRNAs and their adjacent protein-coding genes or pseudo intronic tRNAs and their hosting protein-coding genes.

**Fig 7:**
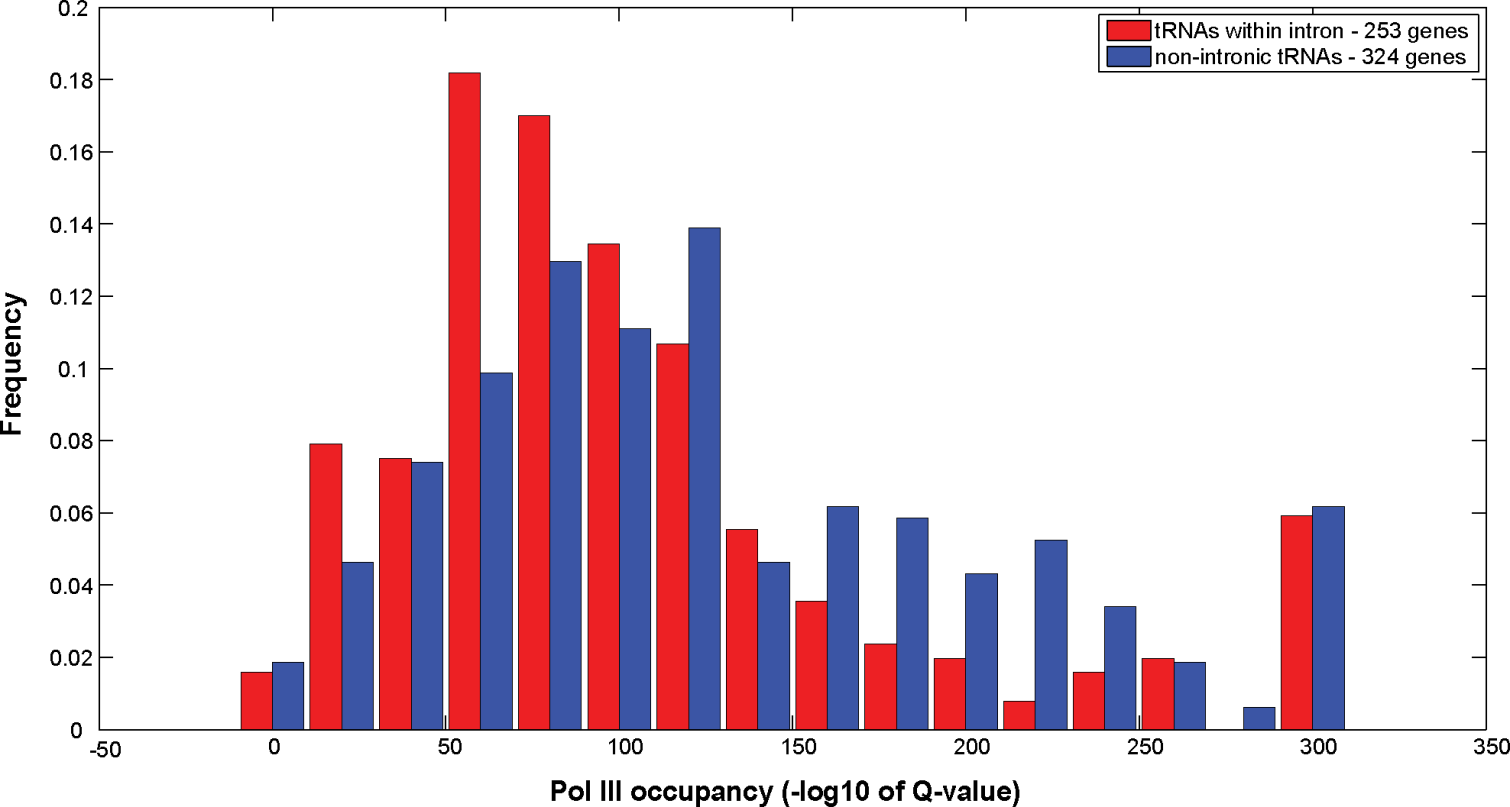
The difference in the PolIII occupancy of intronic and non intronic tRNA genes in *C*. *elegans*. Shown are histograms of PolIII occupancy of young adult worms in intronic (red bars) and non-intronic (blue bars) tRNAs. Occupancy is given in terms of Q-values (-log10 scale). Red bars correspond to intronic tRNAs; blue bars represent non-intronic tRNAs (Wilcoxon rank sum test calculated p-value = 1.31*10^−04^).

**Fig 8:**
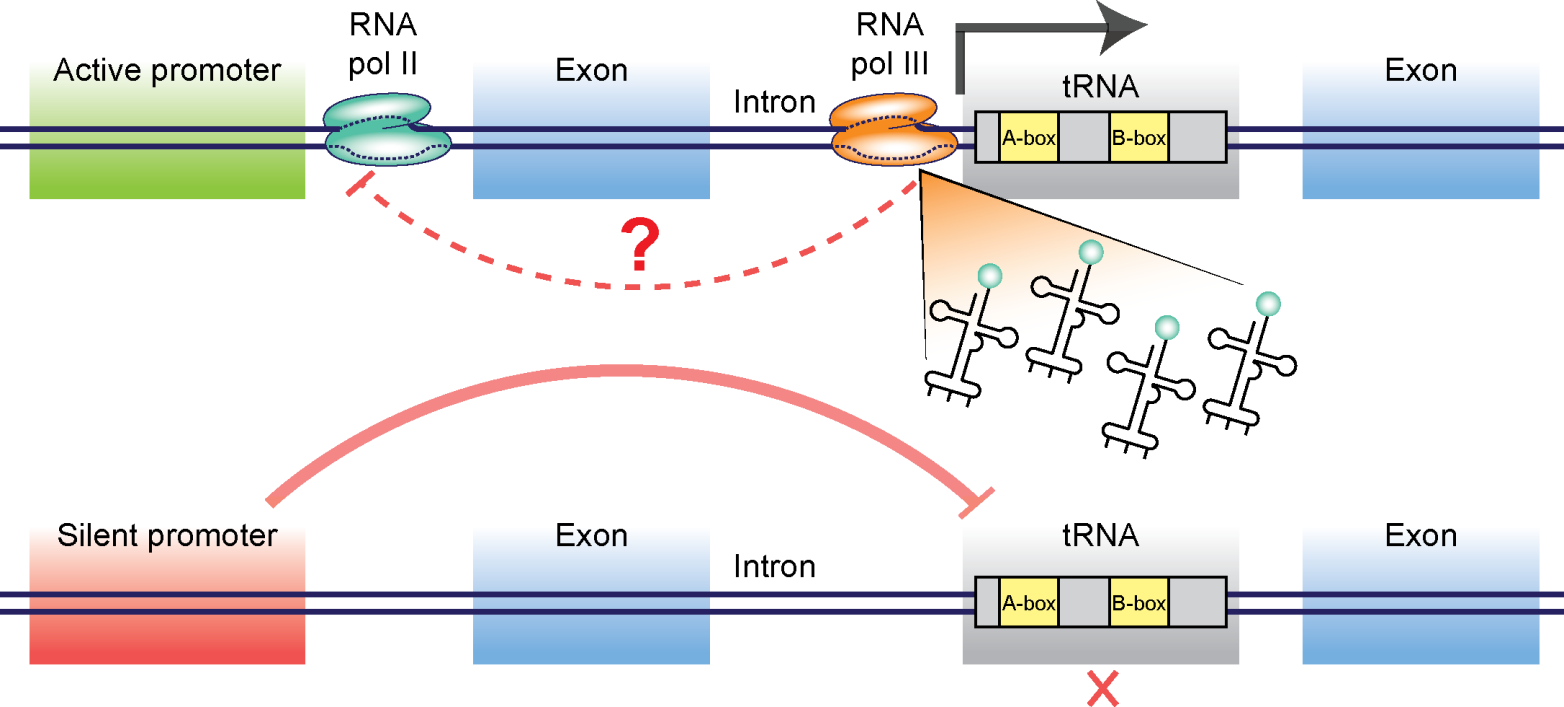
A schematic model for regulating intronic tRNAs by the host gene promoter. tRNA genes are independent entities transcribed by RNA pol III. We showed here that different copies of tRNA are transcribed in a tissue specific manner. One level of regulation can arise from tRNA genes that reside inside intron of a protein coding gene. Shown here are the possible interaction between Pol II gene promoters and Pol III-mediated transcription of intronic tRNAs. Using heat shock promoters we show that promoter repression in cold temperatures can silence the intronic tRNA, and that release of repression in high temperatures enables tRNA expression. The possibility that certain tRNAs can affect the host gene’s expression is also considered (top). Once the gene’s promoter is silenced, tRNA transcription is suppressed (bottom).

Next, we determined whether intronic tRNAs are differentially expressed in comparison with non-intronic tRNAs. We found that intronic tRNAs are characterized by lower Pol III occupancy in comparison with the Pol III occupancy of non-intronic tRNAs (Wilcoxon rank sum test; p-value = 1.31*10^−04^, Fig 7, based on the Chip-Seq experiments that are described in ref [53]). Moreover, intronic tRNAs that reside in the same strand as the hosting protein-coding gene (transcribed in the same orientation) exhibit lower Pol III occupancy in comparison with intronic tRNAs that reside in the opposite strand (Wilcoxon rank sum test; p-value = 0.0042, S6 Fig)). Taken together, our results indicate that the pairing between intronic tRNAs and their hosting protein-coding genes is conserved and affects tRNA expression (see the model in Fig 8).

## Discussion

Sequence-based methods, including micro-arrays, sequencing, and also the recent template and ligation-based method [9,43,60] for tRNA analysis cannot discriminate between identical copies of tRNAs. By utilizing a method for reporting tRNA transcription in live animals, we found that tryptophan tRNA genes, despite their sequence identity, exhibit distinct expression patterns. By analyzing the genomic environment that surrounds tRNAs, we found that the expression of intronic tRNAs is effected by their “host” genes. These results suggest that the association between protein-coding genes and intronic tRNAs allows worms to control tRNA expression temporally and spatially.

We cannot rule out the possibility that mutations in tryptophan tRNAs’ anticodon sequences alter the tRNA expression levels. However, the strong correlation between the measurements that were obtained using the reporter and the Pol III occupancy measurements (that were obtained using animals with wild-type tryptophan tRNA genes) suggests that the expression levels of mutated tryptophan tRNA genes resemble the levels of wild-type tryptophan tRNAs and re-affirms the validity of our reporter gene based method. Moreover, it does not appear that the presence of a wild type copy of the tRNA dramatically changes the tRNA’s levels, as we measured similar expression patterns of the *sup-7* tRNA in worms with a genomic *sup-7* mutation (no functional copy of *sup-7*), and in worms that were injected with a transgene that carried a mutated copy of the *sup-7* gene but contained also a functional genomic copy of the same tRNA.

How does the protein-coding genes support or restrict the expression of tRNAs that reside inside their introns? Is transcription of the protein-coding gene required for transcription of the intron-contained tRNA? Analysis of the expression patterns of 4 protein-coding genes that host 5 intronic tryptophan tRNAs did not reveal such a simple picture: Although *sup-*7 tRNA displays strong read-through activity, the *C03B1*.*2* gene, which hosts *sup-7* in its intron, is very lowly expressed (expression is not detectable using both a GFP reporter and RNA-seq data, S7 Fig, S8 Fig). In fact, 3 out of the 4 “tRNA hosting” protein-coding genes that were examined using fluorescent reporters were expressed at low levels. However, the expression pattern of the only protein-coding gene that we tested that displayed appreciable expression levels, *fli-1*, partially overlapped with the expression pattern of the contained *sup-5* tRNA (S7 Fig). Similarly to the multitude of mechanisms that regulate the transcription of protein-coding genes, it is very probable that manifold control elements that reside outside of tRNA sequences affect tRNA expression [58,61–63]. This complex regulation of tRNA could explain the absence of a simple correlation between the expression of hosting genes and their intron-contained tRNAs. According to our model, the expression of an intronic tRNA can be “licensed” or restricted by proximal promoter elements that affect the local chromatin environment, in ways that either promote or reduce Pol III binding (Fig 8). Analysis of Pol III binding indeed revealed that Pol III differentially binds intronic and non-intronic tRNAs (Fig 7).

It is possible that, in addition to the effects that RNA Pol II genes exert on intronic tRNA expression, intronic tRNAs affect the expression of their host genes (see the model in Fig 8). Indeed, certain *C*. *elegans* genes require a regulatory element that resides within their introns [64]. Our analysis revealed that in *C*. *elegans*, genes that contain tRNAs in their introns exhibit a narrower distribution of expression levels in comparison with all protein-coding genes (S9 Fig), although this analysis does not imply causality.

Over the years, and especially recently, many different roles were discovered for tRNAs, in addition to their traditional role as adaptors that enable the translation of nucleic acid to amino acid code [13,65]. For example, tRNA fragments were shown to inhibit translation initiation [66], and to be intergenerationally inherited [67,68]. Moreover, tRNA-derived microRNAs were found to modulate proliferation and to affect the DNA damage response [69]. It is possible that differential regulation of tRNA expression serves as a means for cell or time-specific synthesis of tRNAs that perform such dedicated regulatory functions.

Similarly to miRtrons (microRNAs that are cut from introns [70]), it is theoretically possible that an intronic tRNA molecule could be spliced from a pre-mRNA sequence. Our genomic analysis showed that only 56% of the intronic tRNAs were transcribed in the same orientation as the mRNA of the host gene (S3 Fig). Thus, splicing of intronic tRNAs from mRNA cannot explain the inclusion of all intronic tRNAs in introns. Nevertheless, it is possible that in some cases (which would be fascinating to study), splicing of tRNAs from introns does take place.

One possible explanation for the evolution of identical tRNA copies that are differentially expressed in metazoans lies in the different translational needs of various tissues. Different tissues develop at different times and rates, and accordingly, there is a variance in the demand for proteins. Tissue-specific expression of specific tRNA genes can thus match the supply of amino acids to the demand for proteins in specific tissues [20,71]. As promoters evolved to drive tissue-specific expression, harnessing these existing elements to express intron-embedded tRNAs at a specific time and place could be a parsimony evolutionary solution.

In aging organisms the mechanisms for precise expression of genes are compromised [72]. The differential temporal expression patterns that we documented for different tRNA genes, and in particular, the decreased neuronal expression that we observed in worms during adulthood (Fig 3), could be related to protein aggregation defects, and especially to the misregulation in translation that is often seen in neurodegenerative diseases. Reduced tRNA levels in aging animals may abrogate co-translational folding by altering the balance between the supply of tRNAs and the demand for mRNAs with specific codon usage, leading to protein misfolding [7,18,73]

In summary, although tRNAs and other Pol III products have been traditionally regarded as “house-keeping genes”, our analysis, and additional accumulating evidence [11,18,20,37,46] suggest that tRNA expression is highly dynamic. The interesting interactions of tRNAs and Pol II protein-coding genes that we revealed here could indicate that Pol II and Pol III also function as co-regulators, and that spatial and temporal gene-specific differential transcription by Pol III could affect an organism’s physiology in health and disease.

## Materials and Methods

### Nematode strains

All *C*. *elegans* strains were maintained and handled as previously described [74]. Transgenic strains were created by microinjecting either N2 or RRS worms. To inject RRS worms, we typically co-injected the gene of interest at 5 ng/μl with 95 ng/μl of 1kb DNA ladder (New England Biolab). Reporter genes were injected into N2 by co-injecting *Pmyo-3*:*mCherry* at 10 ng/μl, 20 ng/μl of the reporter transgene with 70 ng/μl of DNA ladder. To determine the role of the host gene promoter in the wild- type background, N2 worms were injected with concentrations ranging from 10 to 0.5 ng/μl of the host gene that contained the *sup-7* suppression mutation, and DNA ladder to achieve a total concentration of 100 ng/μl DNA. We used the *rol-6 (*su1006*)* gene [75] as a co-injection marker for the indicated lines. Strain numbers and complete genotypes of all new transgenic strains are provided in S3 Table.

The Read-through Reporter Strain (referred to as RRS), which contains the reporter construct *Prps-0*::*mGFP-TAG-mCherry-HA-NLS* strain in a *smg-2 (e2008) background*, was kindly provided by Jason W. Chin [50] (Medical Research Council Laboratory of Molecular Biology, Hills Road, Cambridge, CB2 0QH, U.K). Tryptophan-to-amber suppressor strains were as follows: strain RW2070 (referred to as *sup-7*) and DR478 (*sup-7*B) contain a *sup-7* mutation, strain CB1464 (*sup-5*) and CB2318 (*sup-5*B) contain a *sup-5* mutation, strain CB4227, CB3737, CB4425, CB3909, and CB3874 contain *sup-34*, *sup-29*, *sup-24*, *sup-21*, and *sup-28* mutations, respectively. Strain CB4747, containing the *sup-33* mutation, was kindly provided by Jonathan Hodgkin (University of Oxford, South Parks Road, Oxford, OX1 3QU, UK). Nematodes were cultured on agar plates seeded with *Escherichia coli* at 22°C or at the indicated temperature and handled as described by Sulston and Hodgkin [76]. Crossed strains were constructed by standard genetic approaches, and backcrossed to RRS six times. Brood size was determined by counting the total number of progeny from a single hermaphrodite.

### Microscopy

For all imaging studies, live worms were immobilized on agar slides with 25 mM levamisole to paralyze the worms. Pictures were taken with a 10x (for quantification) or 20x (to identify the expression pattern) lens on a BX63 Olympus microscope and a Retiga 6000 camera. Expression patterns were also determined by confocal microscopy. Quantification of the red or green fluorescence in each worm was performed using ImageJ. Total intensity was calculated by reducing [area x average of 3 background reads] from the intDev. The ratio of red/green fluorescence was calculated for each individual worm. Results from three independent sets of 30 worms were used to calculate the average expression level and SD.

### RNAi assay

The *daf-2* RNAi clone was a kind gift from Ehud Cohen [77]. RNAi knockdown experiments were performed on NGM plates supplemented with 100 ug/mL Ampicillin and 2 mM IPTG to induce dsRNA expression.

### Lifespan prediction assay

Age-synchronized worms were transferred to individual NGM plates and iced for 10 minutes to induce paralysis. Each worm was then imaged on its plate and then the plates were incubated at 20^°^C. Images were analyzed using ImageJ. All pictures were taken on the same day with the same microscope settings. Each plate containing a single worm was labeled with the corresponding picture number and plates were scored for dead worms daily. Lifespans were analyzed at 20^°^C as previously described [56]. Age refers to days following adulthood, and p values were calculated using a linear regression model. Worms were excluded from the analysis when their gonad extruded, or when they desiccated by crawling onto the edge of the housing plate.

### Constructs

All genomic constructs were amplified directly from the corresponding worm strain genome. For example, the transgene containing the *sup-7* suppressor was amplified directly from the RW2070 genome. GFP reporter strain constructs with swapped promoters were built using the Gibson assembly protocol (NEBuilder, New England Biolabs). Unless specified otherwise, as promoters, we amplified the complete intergenomic region upstream of the ATG of the corresponding protein gene.

The primers were as follows:

FWR *C03B1*.*2* with a promoter:

5' CAAACCGAGTGGAAACAATGTTCAAC 3'

FWR *C03B1*.*2* without a promoter:

5' GGGACCCACATGACCATTCCAG 3'

RVS *C03B1*.*2* gene:

5' GCCAGAAAACAATTATTCATAATGACGA 3'

Intron only primers (amplified 900bp upstream and 500bp downstream of the sup-7 gene:

FWR 5' CACGCAGCATTTTCCATGCTAGAG 3'

RVS 5' CGTGTGAGGTCAGCAAAATTCCTATCCG 3'

#### P*hsp-16*.48::*C03B1*.*2* cloning

A PCR fragment containing *C03B1*.*2* from ATG to the end of the 3'UTR (the same as the 'RVS *C03B1*.*2* gene' primer) RW2070 for the *sup-7* construct was cloned using Gibson Assembly (NEB) to replace the coding sequence of the pWD79-2RV (*Phsp-16*.*48*:FLP) vector [78]. P*hsp-16*.48::*C03B1*.*2* containing the wild-type *sup-7* construct was generated using a site-directed-mutagenesis PCR protocol from the P*hsp-16*.48::*C03B1*.*2* template.

#### *Pmyo-3*::*C03B1*.*2* and *Punc-122*::*C03B1*.*2* cloning

We used Gibson assembly to generate a linear fragment containing the *myo-3* or *unc-122* promoter fused to the *C03B1*.*2* gene from the RW2070 strain (which contains the *sup-7* amber read-through mutation, similarly to the P*hsp-16*.48::*C03B1*.*2* cloning). All constructs were verified by sequencing. All "*sup*" mutations were validated by XBaI restriction of the PCR products.

### Data sources

The tRNA gene copy numbers of all analyzed species were downloaded from the Genomic tRNA Database (http://lowelab.ucsc.edu/GtRNAdb)[79].

The genomic location of protein-coding genes and tRNAs of all analyzed species were downloaded from the RefSeq Genes Track at the UCSC website (http://genome.ucsc.edu/). The locations of introns were calculated based on the start and end positions of the transcripts' exons. The genomic locations of tRNAs of all the analyzed species were downloaded from the tRNAs Genes Track at UCSC, which displays tRNA genes predicted by using tRNAscan-SE v.1.23.

For each individual species, the protein coding genes and tRNA coordinates were downloaded from the same assembly (*C*. *elegans*–WS190/ce6; *C*. *brenneri*–WUGSC 6.0.1/caePb2; *C*. *briggsae*—WUGSC 1.0/cb3; *C*. *japonica*- WUGSC 3.0.2/ caeJap1; *C*. *remanei*—WUGSC 15.0.1/caeRem3; *P*. *pacificus*—WUGSC 5.0/priPac1).

Pol III occupancy data in terms of the Q-values of significance of the binding activity for young adult nematode were downloaded from modENCODE (Snyder_N2_POLIII_YA; modENCODE_4034) [52,53]. The ChIP-seq data generated by this experiment were analyzed using the PeakSeq peak-calling algorithm [80]. H3K4me3 MA2C scores for young adult nematodes were downloaded from modENCODE (Lieb_N2_H3K4me3_YA; modENCODE_3552); H3K27ac MACS scores for young adult nematodes were downloaded from modENCODE (Lieb_H3K27ac_YA; modENCODE_3921) [81].

### Conservation of the tRNAs’ genomic location among nematodes

In this analysis we examined whether the localization of a given tRNA type within the transcript of a specific protein-coding gene is conserved among nematodes. We first looked for all the occurrences of functional tRNA genes within transcripts of protein-coding genes in *C*. *elegans*. Then, we characterized each such occurrence as a pair of a specific anticodon and specific protein-coding gene. If more than one tRNA gene was assigned to the same transcript, the number of pairs was determined by the number of the different tRNA types (anticodons). Overall, we detected 208 such pairs in *C*. *elegans*, associated with 188 unique protein-coding genes (in some cases two or more tRNA types (anticodons) reside in the same protein-coding genes). Next, we repeated the analysis for four additional nematode species (*C*. *brenneri*, *C*. *briggsae*, *C*. *japonica*, and *C*. *remanei*), and for the distant *Pristionchus pacificus* species. Then, the conservation of tRNA types within transcripts was defined as the fraction of the existing anticodon-protein-coding gene pairs in *C*. *elegans* that are conserved among each of the five additional species, while excluding cases in which the *C*. *elegans* protein-coding gene has no orthologue in the other examined species.

In order to assess to what extent the conservation of a given tRNA type within a specific protein-coding gene, among the nematode species, was derived from global conservation in the genome organization, we also searched for the closest (non-overlapping) protein-coding genes of the non-intronic tRNA gene (the closest protein-coding gene both upstream and downstream relative to the 5’ and 3’ of the tRNA). We then separately calculated for each set of upstream or downstream neighboring genes the conservation of the identity of the adjacent protein-coding genes of a given anticodon among the nematodes. Here too, we excluded cases in which the *C*. *elegans* protein-coding gene had no ortholog in other examined species. We repeated these analyses separately for the sets of pseudo tRNA genes.

### Mean FPKM expression values of *the C*. *elegans* protein-coding genes

The mean FPKM expression values of all the genes of *C*. *elegans* were kindly generated for us by Paul Davis (WormBase staff). The mean value of a given gene at a given stage indicates the mean of the *n* RNASeq FPKM expression values for this gene at this life-stage of all wild-type control samples in the WormeBase SRA ("*n*" may vary between both the genes and the life stages).

## Supporting Information

S1 Fig Identical tryptophan tRNAs alleles are expressed similarly. (A) Representative mCherry expression of young adults population of *sup-5 and sup-5 B*. (B) Quantification of total worm mCherry fluorescence. All strains were analyzed when the worms were the same age (young adults), using the same exposure parameters. Shown are averages of means, ± SEM, normalized to the expression levels detected in the *sup-7* strain. Differences between the same “sup” strains were not significant (sup-7 vs. sup-7B, p-value = 0.1159, sup-5 vs. sup-5B, p-value = 0.3051), whereas for all other comparison p-value<0.001. Data for the strains *sup-5* and *sup-7* are the same as in Fig 2.
(TIF)

S2 Fig Comparison of tRNA expression measured by the read-through reporter and measurements of H3K4me3 and H3K27ac histone modifications. (A) Maximal H3K4me3 MA2C scores upstream of the tRNA genes (-500 to -100 compared to the first nucleotide of the mature tRNAs) were plotted against total worm mCherry fluorescence measurements of all "sup" strains. (B) Maximal H3K4me3 MA2C score upstream of the tRNA genes (-500 to -100 compared to the first nucleotide of the mature tRNAs) were plotted against PolIII occupancy tRNAs in young adult worms. Occupancy is given in terms of Q-values (-log10 scale). (C) Profile of H3K4me3 modification in the vicinity of tRNA genes in young adult worms. All tRNA genes were aligned according to their TSSs, and the regions of 1000 bp upstream and downstream of the first mature nucleotide are shown on the x axis. The y axis shows the averaged H3K4me3 MA2C scores as a function of distance of all the 609 tRNAs in the genome of C. elegans. (D) Profile of H3K4me3 modification in the vicinity of 12 tryptophan tRNA genes in young adult worms. All tRNA genes were aligned according to their TSSs, and the regions of 1000 bp upstream and downstream of the first mature nucleotide are shown on the x axis. The y axis shows the H3K4me3 MA2Cscore as a function of distance. (E) Maximal H3K27ac MACS scores in the vicinity of tRNA genes (-200 to +200 compared to the first nucleotide of the mature tRNAs) were plotted against total worm mCherry fluorescence measurements of all "sup" strains. (F) Maximal H3K27ac MACS scores in the vicinity of tRNA genes (-200 to +200 compared to the first nucleotide of the mature tRNAs) were plotted against PolIII occupancy tRNAs in young adult worms. Occupancy is given in terms of Q-values (-log10 scale). (G) Profile of H3K27ac modification in the vicinity of tRNA genes in young adult worms. All tRNA genes were aligned according to their TSSs, and the regions of 1000 bp upstream and downstream of the first mature nucleotide are shown on the x axis. The y axis shows the averaged H3K27ac MACS scores as a function of distance of all the 609 tRNAs in the genome of C. elegans. (H) Profile of H3K27ac modification in the vicinity of 12 tryptophan tRNA genes in young adult worms. All tRNA genes were aligned according to their TSSs, and the regions of 1000 bp upstream and downstream of the first mature nucleotide are shown on the x axis. The y axis shows the H3K27ac MACS scores as a function of distance.
(TIF)

S3 Fig Genome-wide characterization of tRNA genomic localizations. Shown are the percentages of tRNA genes in *C*. *elegans* that reside within introns of protein-coding genes (in green if both the tRNA and the protein-coding gene are located on the same strand; in red in case of opposite strands, blue denotes the percentage of tRNAs not localized within introns). The tRNA genes are divided into three subsets: all the tRNAs (left panel), pseudo tRNAs (middle panel), and functional tRNAs (right panel).
(TIF)

S4 Fig Host gene promoter affects the expression pattern of the contained tRNA. Analysis of the *sup-7* neuronal expression pattern in control (*sup-7* worms) and in transgenic worms injected with the *C03B1*.*2* gene with or without promoter. The expression pattern was similar to control worms only in the presence of the host gene promoter (n = 15 pooled data)
(TIF)

S5 Fig The higher conservation of tRNAs and their hosting genes compared with that of tRNAs and their adjacent genes among nematode species is not governed by the distance between the tRNAs and the protein-coding genes. To determine whether the lower degree of conservation in the pairing between tRNAs and the adjacent protein-coding genes stems from the fact that these entities are more distant, on average, we sorted all the distances between individual non-intronic tRNAs and their nearest up-stream protein-coding gene neighbors. We then calculated the conservation for 20 windows; each contains 40 distances (the overlap between two adjacent windows is ~57%). The number of orthologous protein-coding genes associated with each such window vary from ~17 to ~33 (median = 25), depending on the examined species. Each dot corresponds to one of the 20 windows; the x-axis denotes the median of the distances in each window, whereas the y-axis depicts the calculated conservation of each window. The red circles indicate the extent of the conservation of specific anticodons within specific transcripts (i.e., distance = 0). In order to determine whether the observed extent of conservation of intronic tRNAs deviates from the expected conservation, we used the third degree polynomial, and extrapolated the value for distance = 0. The values of a third degree polynomial evaluated at x = 0 (i.e., the median distance of 0) are below the calculated conservation of the intronic-tRNAs (*C*. *remanei*: the extrapolated value at x = 0 is 0.2415 ± 0.0959; *C*. *briggsae*: the extrapolated value at x = 0 is 0.2508 ± 0.0949; *C*. *brenneri*: the extrapolated value at x = 0 is 0.3786 ± 0.0907; *C*. *japonica*: the extrapolated value at x = 0 is 0.1107 ± 0.0873).
(TIF)

S6 Fig Differences between PolIII occupancy of intronic tRNAs in *C*. *elegans* that reside on the same strand as their hosting gene and intronic tRNAs that are located on the opposite strand of their hosting gene. Histograms of PolIII occupancy are given in terms of Q-values (-log10 scale). Blue bars correspond to intronic tRNAs (the same orientation); pale blue bars represent intronic tRNAs (the opposite orientation). S2 Table denotes the p-value of a two-sided Wilcoxon rank sum test for comparisons between the indicated sets of tRNAs.
(TIF)

S7 Fig Expression of tryptophan tRNA hosting genes. Left panel: Representative image of N2 bristol worms injected with the indicated transcriptional GFP reporter. Right panel: Representative image of mCherry read-through expression of the corresponding amber suppressor strains (the same stage). Other than the *fli-1* reporter, all other GFP reports were indistinguishable from the background gut auto-fluorescence.
(TIF)

S8 Fig Time-specific expression of tryptophan tRNA hosting genes. Shown are the mean FPKM expression values of the indicated genes throughout different life stages, representing all wild-type samples of WormBase SRA ("n" may vary between both the genes and the life stages).
(TIF)

S9 Fig Intronic tRNAs hosting genes have a narrower distribution of gene expression levels compared with the general protein-coding gene population. Shown are the distributions of the mean FPKM expression values of different gene sets throughout different life stages ("n" may vary between both the genes and the life stages). Each blue curve corresponds to a set of 186 random protein-coding genes of *C*. *elegans;* 500 such sets are shown. The red lines show the distribution of the mean FPKM expression values of the 186 tRNA-hosting genes in *C*. *elegans*. P-values for the different stages analyzed are: Embryo = 0.002, L1 = 0.002, L2 = 0.002, L3 = 0.002, L4 = 0.046, Adult = 0.066.
(TIF)

S1 Table Summary of tryptophan tRNA hosting gene expression patterns. (XLSX)

S2 Table Summary of the two-sided Wilcoxon rank sum test for comparisons between the pol III occupancy of the indicated sets of tRNAs. (XLSX)

S3 Table List of the strain numbers and complete genotypes of new transgenic strains described in this work. (XLSX)
