# Supplementary figures and images for: Tissue- and Time-Specific Expression of Otherwise Identical tRNA Genes
Source: PLoS Genet. 2016 Aug 25;12(8):e1006264. doi: 10.1371/journal.pgen.1006264 (PMC4999229; doi:10.1371/journal.pgen.1006264)

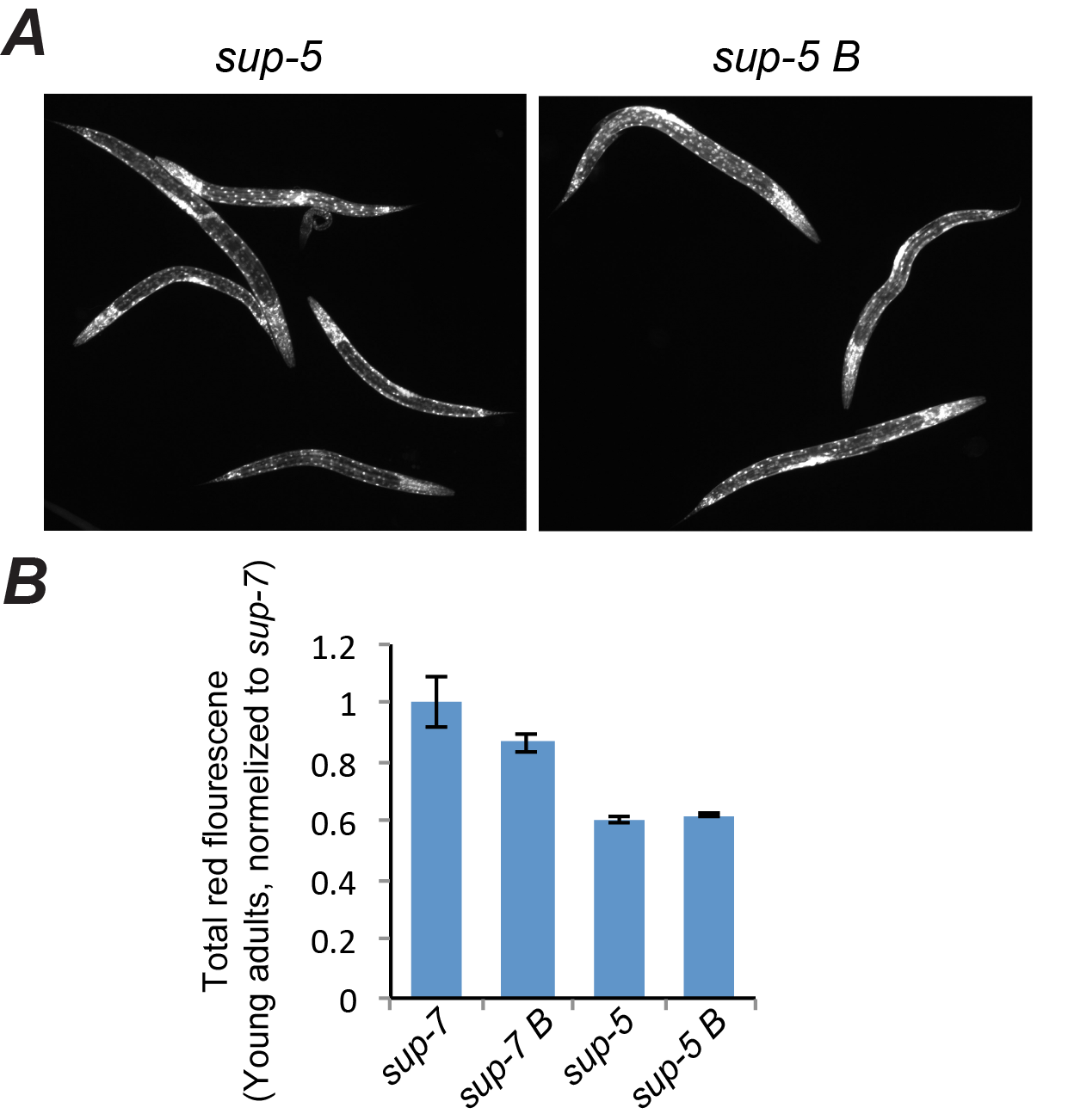

Supplement: S1 Fig — (A) Representative mCherry expression of young adults population of sup-5 and sup-5 B. (B) Quantification of total worm mCherry fluorescence. All strains were analyzed when the worms were the same age (young adults), using the same exposure parameters. Shown are averages of means, ± SEM, normalized to the expression levels detected in the sup-7 strain. Differences between the same “sup” strains were not significant (sup-7 vs. sup-7B, p-value = 0.1159, sup-5 vs. sup-5B, p-value = 0.3051), whereas for all other comparison p-value<0.001. Data for the strains sup-5 and sup-7 are the same as in Fig 2. (TIF) [file pgen.1006264.s001.tif]

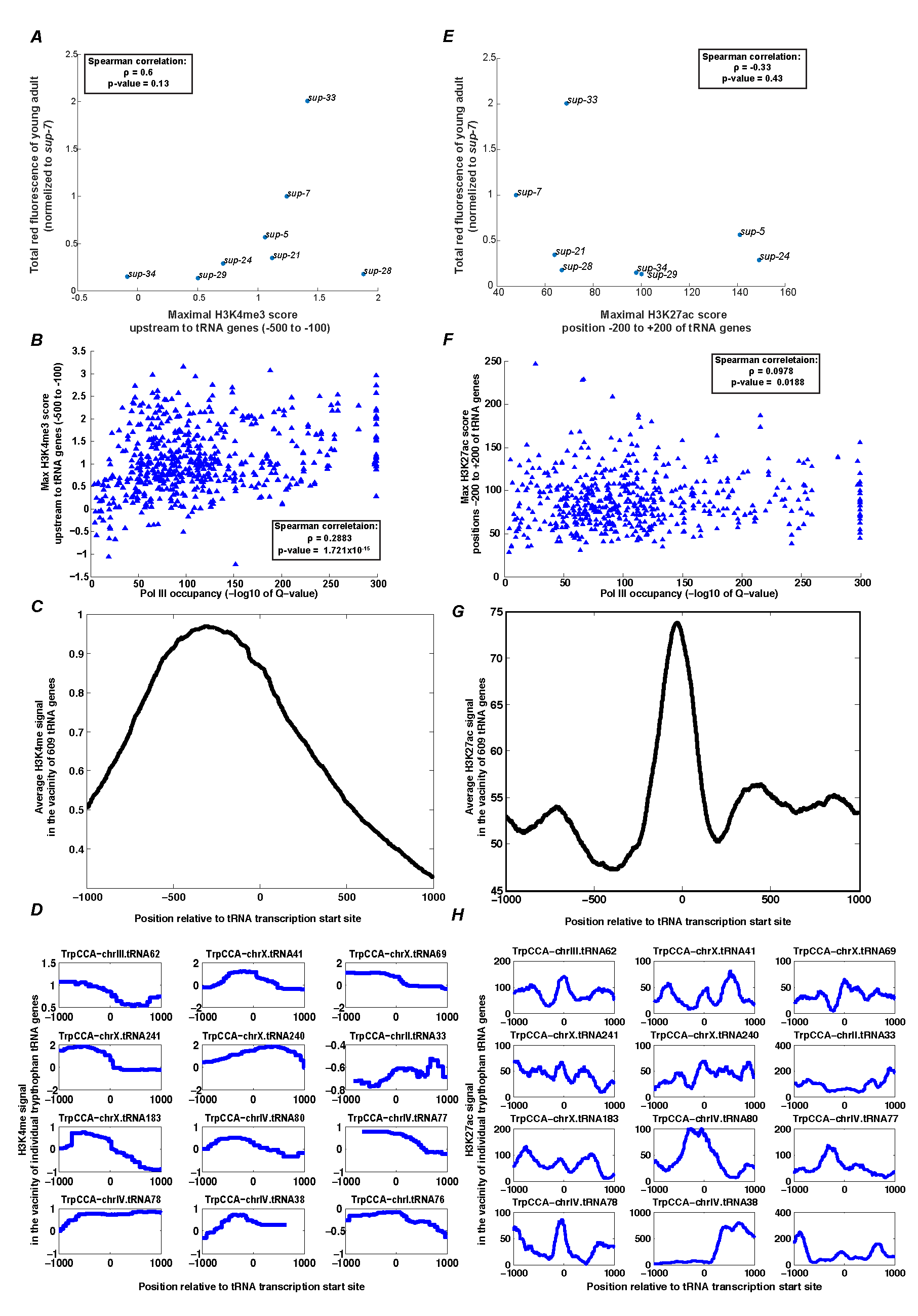

Supplement: S2 Fig — (A) Maximal H3K4me3 MA2C scores upstream of the tRNA genes (-500 to -100 compared to the first nucleotide of the mature tRNAs) were plotted against total worm mCherry fluorescence measurements of all "sup" strains. (B) Maximal H3K4me3 MA2C score upstream of the tRNA genes (-500 to -100 compared to the first nucleotide of the mature tRNAs) were plotted against PolIII occupancy tRNAs in young adult worms. Occupancy is given in terms of Q-values (-log10 scale). (C) Profile of H3K4me3 modification in the vicinity of tRNA genes in young adult worms. All tRNA genes were aligned according to their TSSs, and the regions of 1000 bp upstream and downstream of the first mature nucleotide are shown on the x axis. The y axis shows the averaged H3K4me3 MA2C scores as a function of distance of all the 609 tRNAs in the genome of C. elegans. (D) Profile of H3K4me3 modification in the vicinity of 12 tryptophan tRNA genes in young adult worms. All tRNA genes were aligned according to their TSSs, and the regions of 1000 bp upstream and downstream of the first mature nucleotide are shown on the x axis. The y axis shows the H3K4me3 MA2Cscore as a function of distance. (E) Maximal H3K27ac MACS scores in the vicinity of tRNA genes (-200 to +200 compared to the first nucleotide of the mature tRNAs) were plotted against total worm mCherry fluorescence measurements of all "sup" strains. (F) Maximal H3K27ac MACS scores in the vicinity of tRNA genes (-200 to +200 compared to the first nucleotide of the mature tRNAs) were plotted against PolIII occupancy tRNAs in young adult worms. Occupancy is given in terms of Q-values (-log10 scale). (G) Profile of H3K27ac modification in the vicinity of tRNA genes in young adult worms. All tRNA genes were aligned according to their TSSs, and the regions of 1000 bp upstream and downstream of the first mature nucleotide are shown on the x axis. The y axis shows the averaged H3K27ac MACS scores as a function of distance of all the 609 tRNAs in the genome of C. [file pgen.1006264.s002.tif]

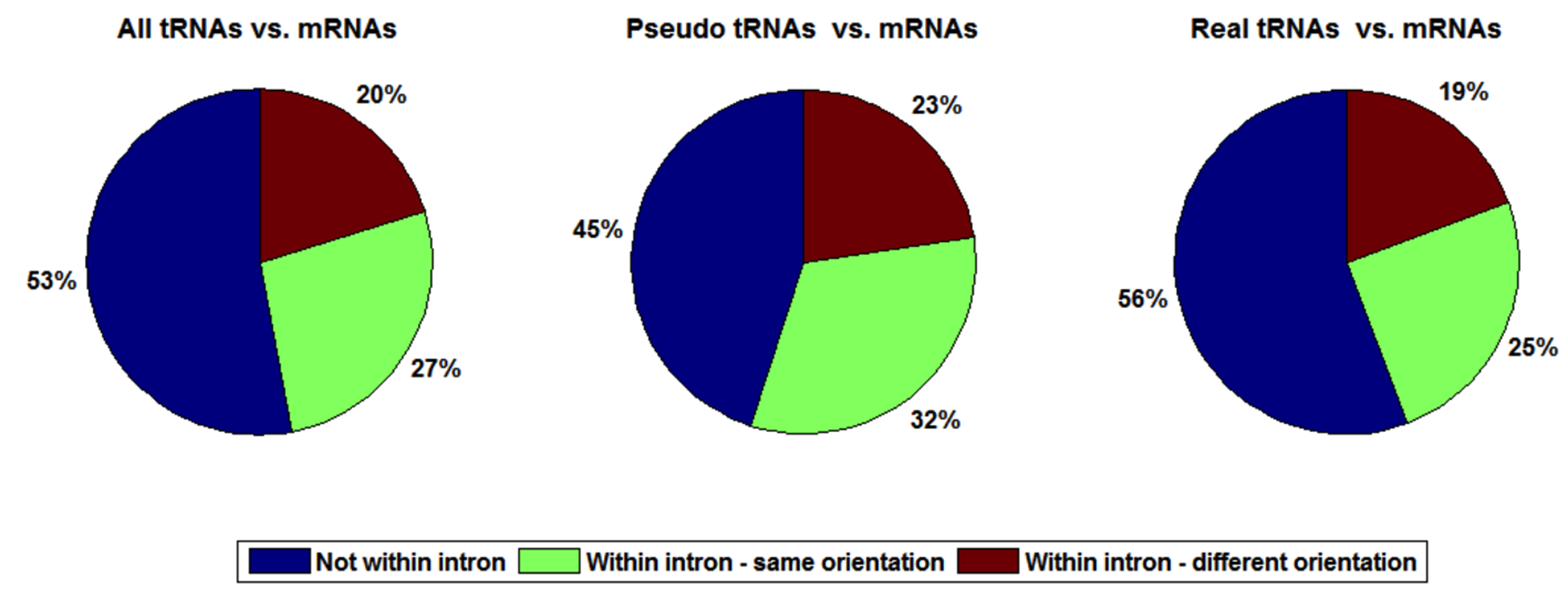

Supplement: S3 Fig — Shown are the percentages of tRNA genes in C. elegans that reside within introns of protein-coding genes (in green if both the tRNA and the protein-coding gene are located on the same strand; in red in case of opposite strands, blue denotes the percentage of tRNAs not localized within introns). The tRNA genes are divided into three subsets: all the tRNAs (left panel), pseudo tRNAs (middle panel), and functional tRNAs (right panel). (TIF) [file pgen.1006264.s003.tif]

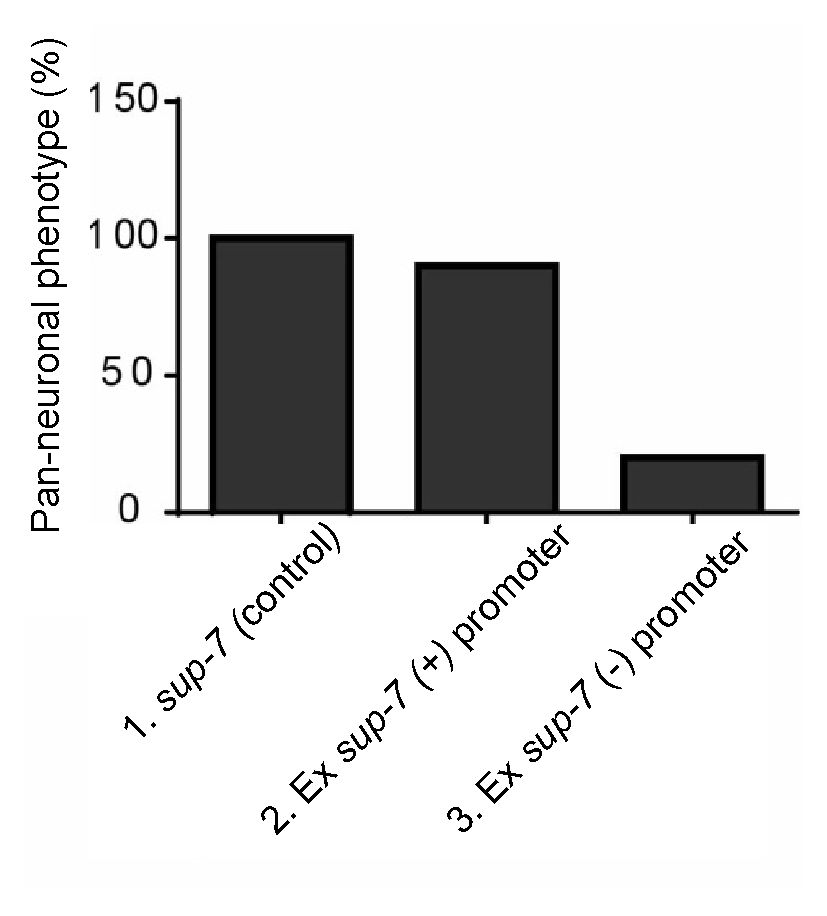

Supplement: S4 Fig — Analysis of the sup-7 neuronal expression pattern in control (sup-7 worms) and in transgenic worms injected with the C03B1.2 gene with or without promoter. The expression pattern was similar to control worms only in the presence of the host gene promoter (n = 15 pooled data) (TIF) [file pgen.1006264.s004.tif]

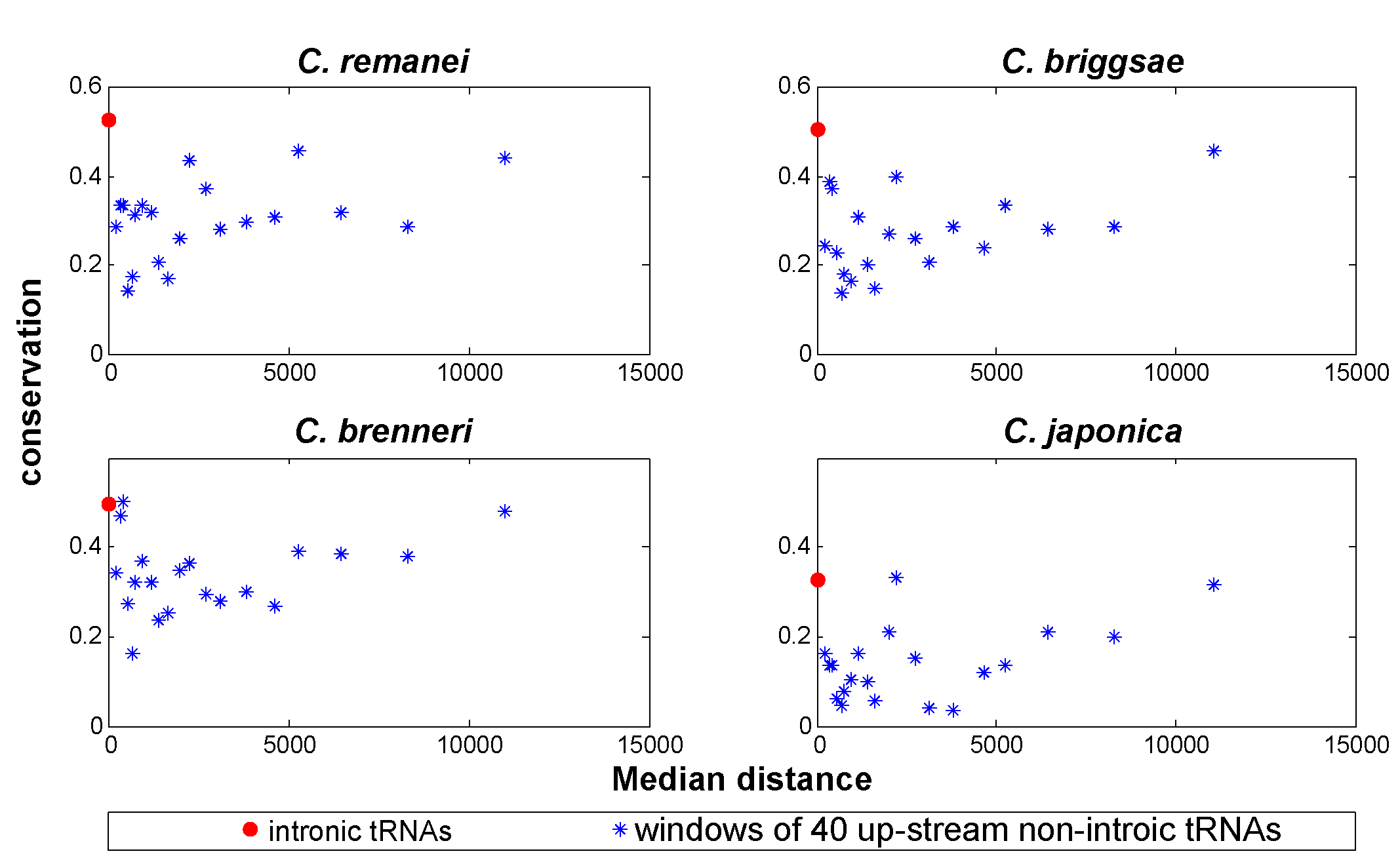

Supplement: S5 Fig — To determine whether the lower degree of conservation in the pairing between tRNAs and the adjacent protein-coding genes stems from the fact that these entities are more distant, on average, we sorted all the distances between individual non-intronic tRNAs and their nearest up-stream protein-coding gene neighbors. We then calculated the conservation for 20 windows; each contains 40 distances (the overlap between two adjacent windows is ~57%). The number of orthologous protein-coding genes associated with each such window vary from ~17 to ~33 (median = 25), depending on the examined species. Each dot corresponds to one of the 20 windows; the x-axis denotes the median of the distances in each window, whereas the y-axis depicts the calculated conservation of each window. The red circles indicate the extent of the conservation of specific anticodons within specific transcripts (i.e., distance = 0). In order to determine whether the observed extent of conservation of intronic tRNAs deviates from the expected conservation, we used the third degree polynomial, and extrapolated the value for distance = 0. The values of a third degree polynomial evaluated at x = 0 (i.e., the median distance of 0) are below the calculated conservation of the intronic-tRNAs (C. remanei: the extrapolated value at x = 0 is 0.2415 ± 0.0959; C. briggsae: the extrapolated value at x = 0 is 0.2508 ± 0.0949; C. brenneri: the extrapolated value at x = 0 is 0.3786 ± 0.0907; C. japonica: the extrapolated value at x = 0 is 0.1107 ± 0.0873). (TIF) [file pgen.1006264.s005.tif]

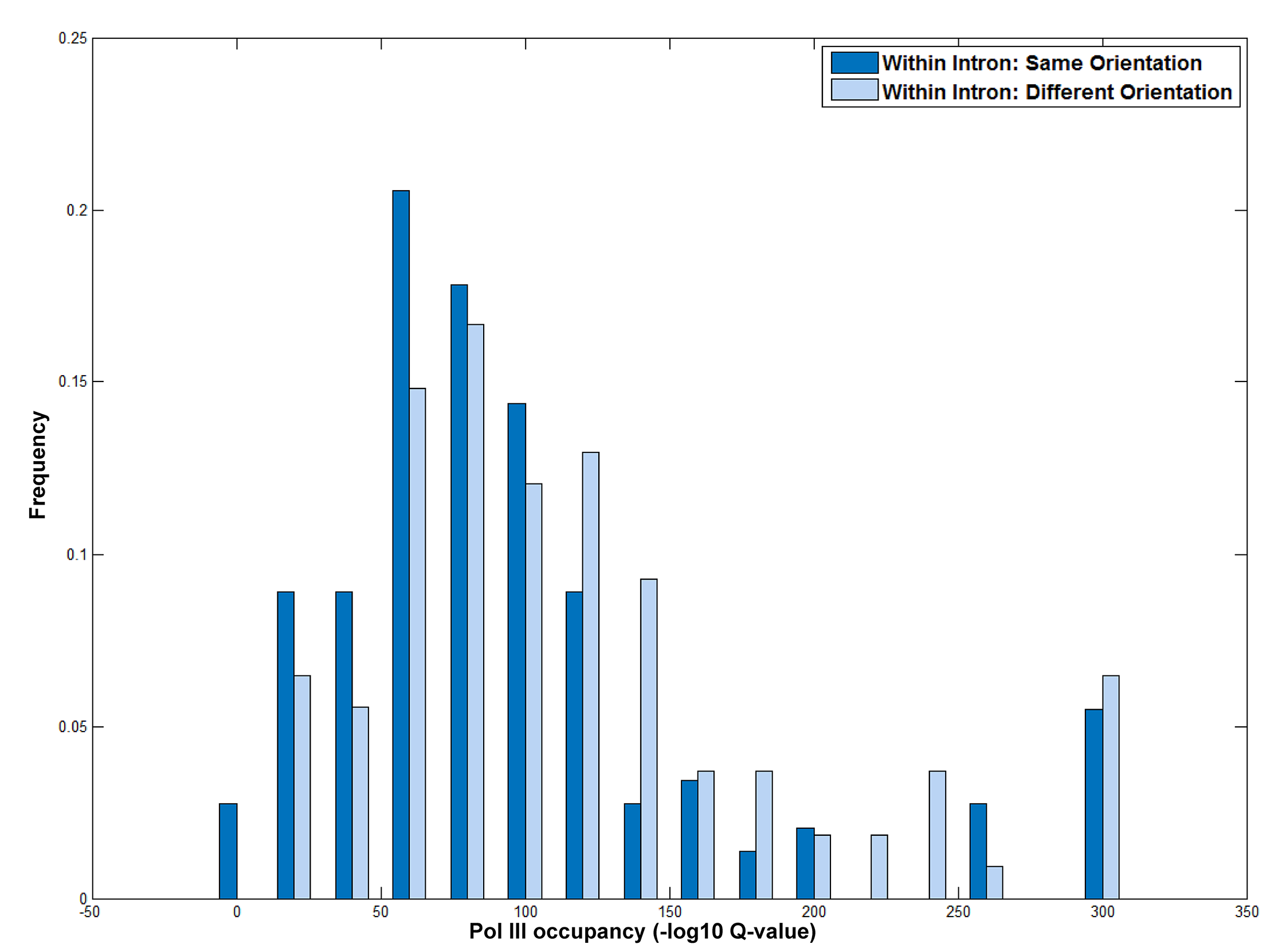

Supplement: S6 Fig — Histograms of PolIII occupancy are given in terms of Q-values (-log10 scale). Blue bars correspond to intronic tRNAs (the same orientation); pale blue bars represent intronic tRNAs (the opposite orientation). S2 Table denotes the p-value of a two-sided Wilcoxon rank sum test for comparisons between the indicated sets of tRNAs. (TIF) [file pgen.1006264.s006.tif]

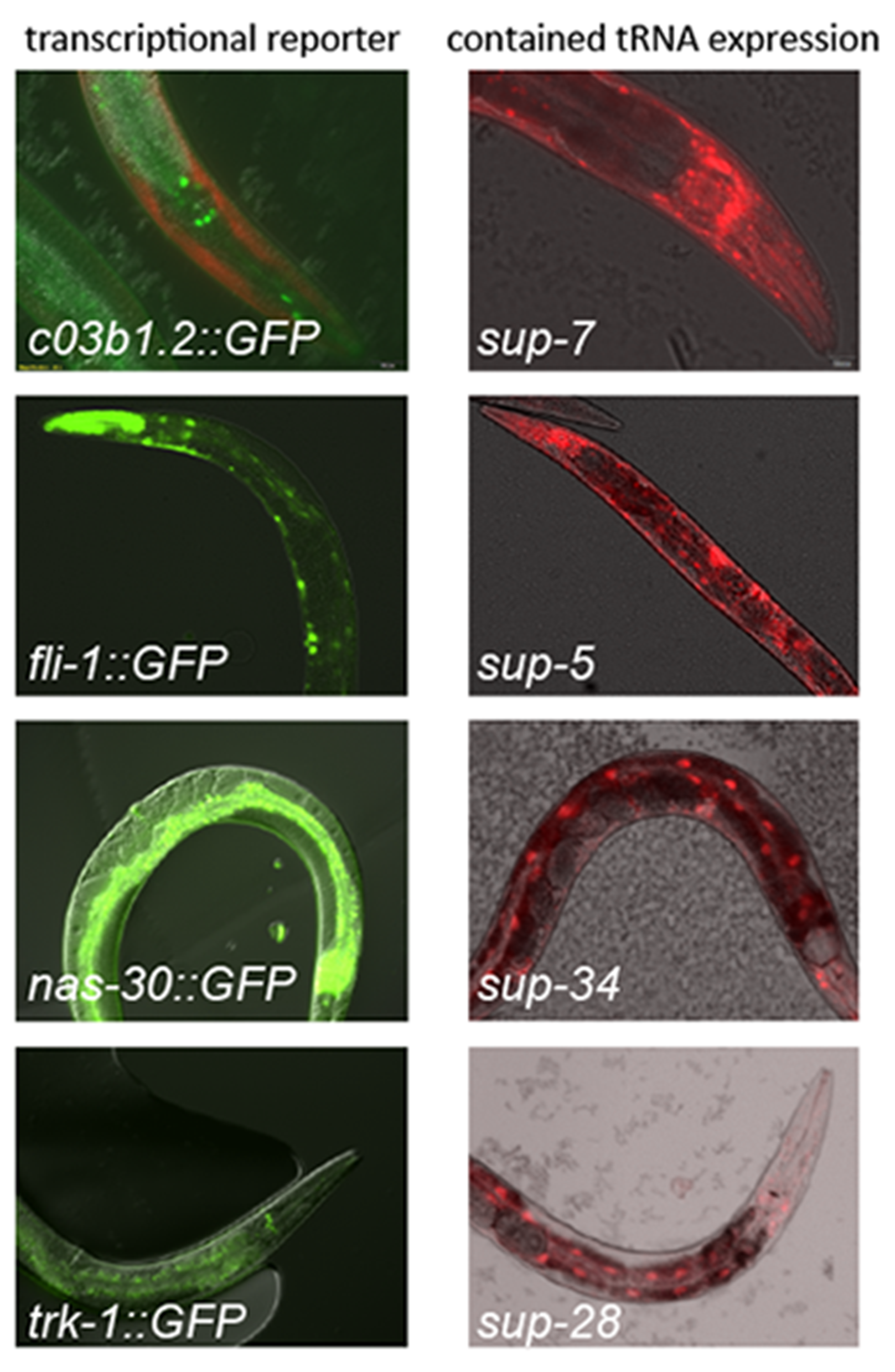

Supplement: S7 Fig — Left panel: Representative image of N2 bristol worms injected with the indicated transcriptional GFP reporter. Right panel: Representative image of mCherry read-through expression of the corresponding amber suppressor strains (the same stage). Other than the fli-1 reporter, all other GFP reports were indistinguishable from the background gut auto-fluorescence. (TIF) [file pgen.1006264.s007.tif]

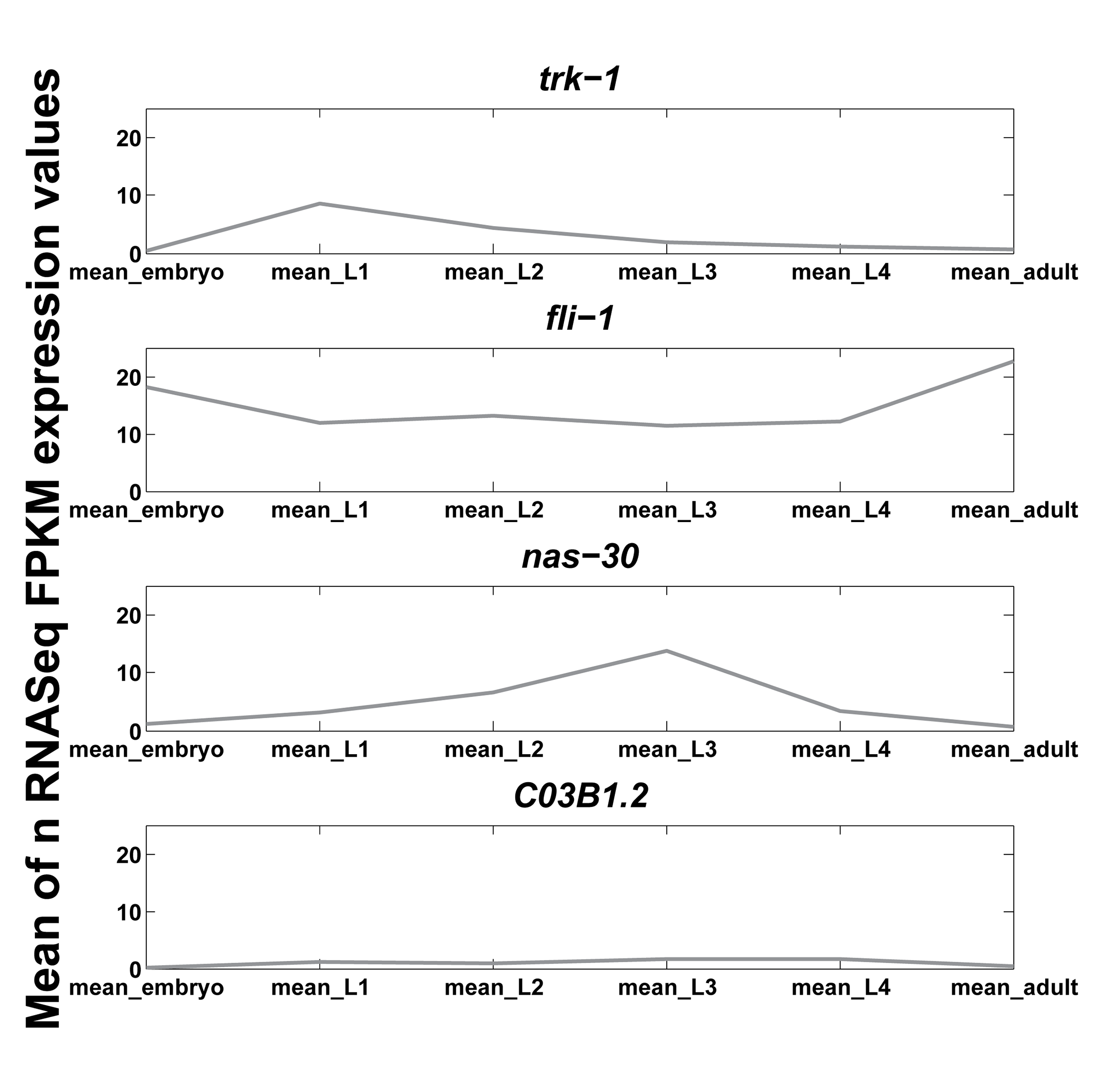

Supplement: S8 Fig — Shown are the mean FPKM expression values of the indicated genes throughout different life stages, representing all wild-type samples of WormBase SRA ("n" may vary between both the genes and the life stages). (TIF) [file pgen.1006264.s008.tif]

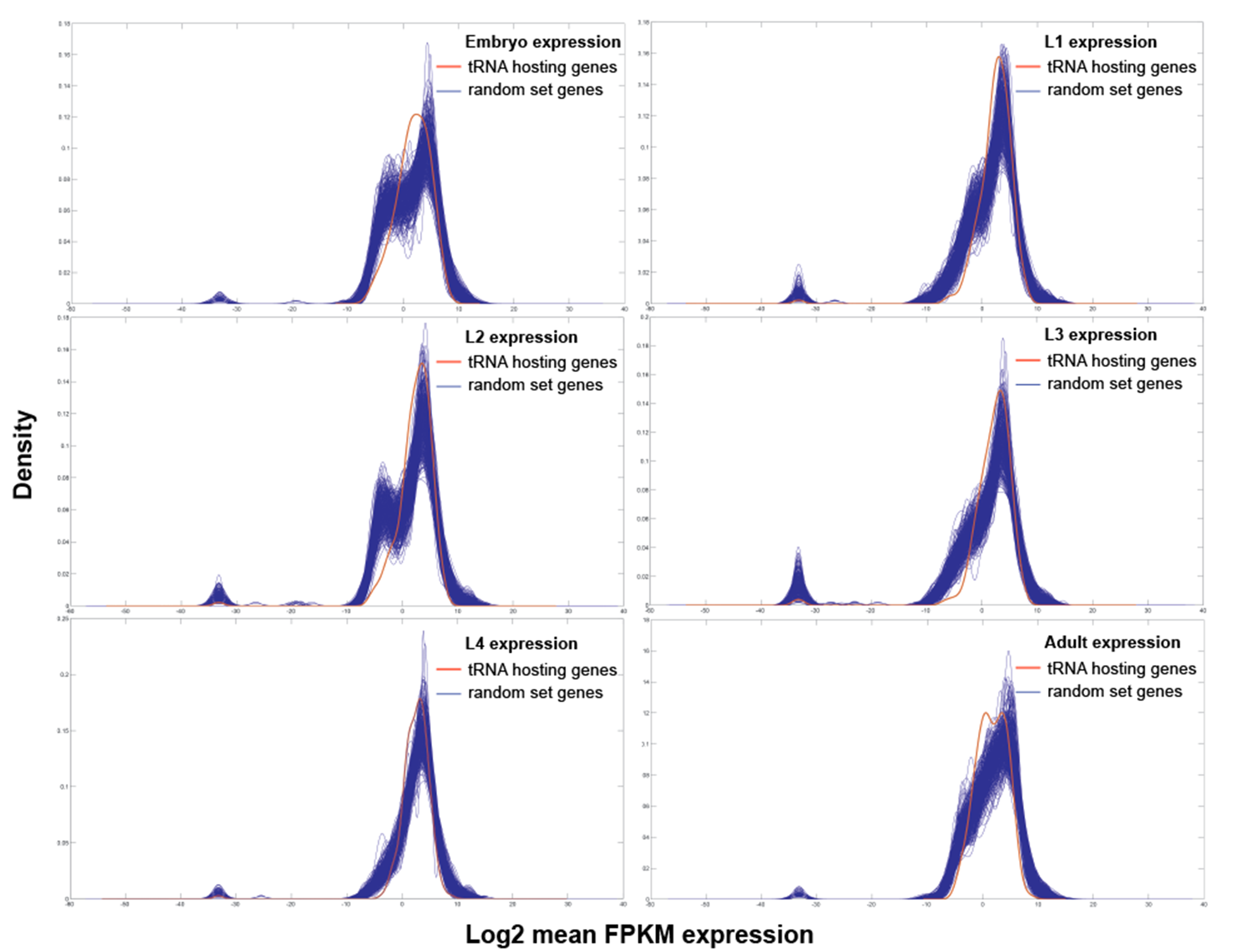

Supplement: S9 Fig — Shown are the distributions of the mean FPKM expression values of different gene sets throughout different life stages ("n" may vary between both the genes and the life stages). Each blue curve corresponds to a set of 186 random protein-coding genes of C. elegans; 500 such sets are shown. The red lines show the distribution of the mean FPKM expression values of the 186 tRNA-hosting genes in C. elegans. P-values for the different stages analyzed are: Embryo = 0.002, L1 = 0.002, L2 = 0.002, L3 = 0.002, L4 = 0.046, Adult = 0.066. (TIF) [file pgen.1006264.s009.tif]
